# Hepatocyte hedgehog signaling controls ferroptosis to alleviate aging-related organ dysfunction

**DOI:** 10.1172/jci.insight.207066

**Published:** 2026-05-19

**Authors:** Ji Hye Jun, Rajesh Kumar Dutta, Soon-Woo Cho, Rui Yao, Seh Hoon Oh, Zhi Li, Kuo Du, David S. Umbaugh, Nanchao Wang, Yirui Xu, Jingting Li, Lingyan Shi, Jen-Tsan Chi, Junjie Yao, Anna Mae Diehl

**Affiliations:** 1 Division of Gastroenterology, Department of Medicine, Duke University, Durham, North Carolina, USA.; 2 Division of Life Science, College of Natural Sciences, Gyeongsang National University, Jinju, South Korea.; 3 Department of Biomedical Engineering, Duke University, Durham, North Carolina, USA.; 4 Shu Chien-Gene Lay Department of Bioengineering, University of California, San Diego, California, USA.; 5 Department of Molecular Genetics and Microbiology, Duke University, Durham, North Carolina, USA.

**Keywords:** Aging, Hepatology, Metabolism, Cellular senescence, Signal transduction

## Abstract

Aging drives systemic metabolic dysfunction (SMD) and increases the risk of chronic illnesses such as metabolic dysfunction–associated steatotic liver disease (MASLD) and chronic kidney disease (CKD). However, mechanisms that connect aging to multiorgan deterioration are poorly understood. In this study, we identify hepatocyte Hedgehog signaling as a central regulator of ferroptosis. Using mice with hepatocyte-specific deletion of Smoothened (Smo), a key Hedgehog pathway component, we show that loss of hepatocyte Hedgehog signaling induces ferroptotic stress, lipid peroxidation, and cellular senescence. These changes were sufficient to cause spontaneous MASLD and to trigger secondary kidney injury. Smo deletion also disrupted systemic iron balance, increased hepatocyte production of angiotensinogen, and reduced liver perfusion. Similar responses (iron dysregulation, vascular dysfunction, and reduced Hedgehog signaling) were observed in patients with MASLD and advanced fibrosis. Inhibition of ferroptosis with ferrostatin-1 reversed hepatocyte senescence, restored hepatic blood flow, and improved both liver and kidney injury in Smo-deficient mice. Overall, these findings show that hepatocyte Hedgehog signaling preserves liver homeostasis by restraining ferroptotic stress and coordinating iron-dependent vasoactive pathways. The results reveal an unrecognized aging-related communication axis between the liver and kidney and identify the Hedgehog/ferroptosis pathway as a promising therapeutic target for age-associated metabolic diseases.

## Abstract 2

Hepatocytes determine rate of organ aging during metabolic stress

## Graphical abstract

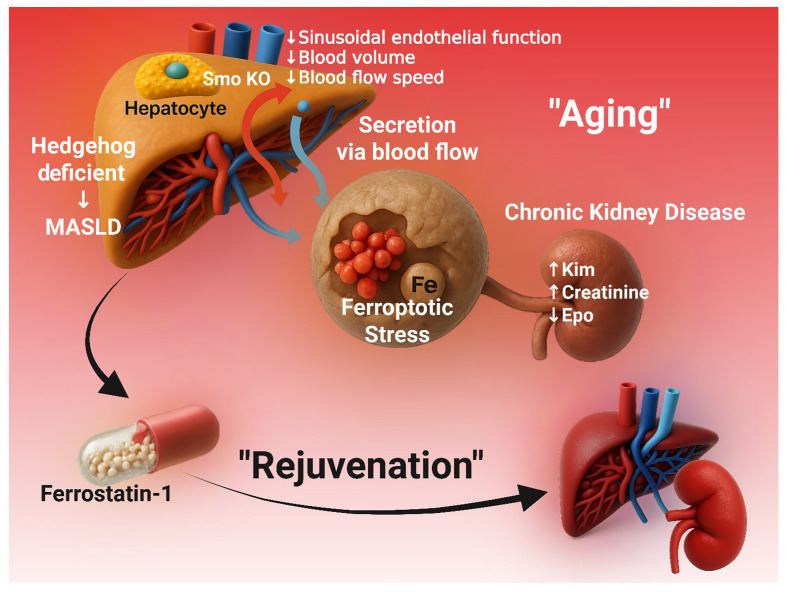

## Introduction

Older age increases the likelihood of developing systemic metabolic dysfunction (SMD) (1). The reasons that metabolic homeostasis deteriorates with age, and why some individuals are more susceptible than others, are not fully understood. This is an important gap in knowledge, because many life-threatening disorders associated with SMD, including obesity, type 2 diabetes, neurodegenerative diseases, chronic kidney disease (CKD), cardiac dysfunction, and metabolic dysfunction–associated steatotic liver disease (MASLD), are now thought to result from accelerated tissue aging (2, 3). These conditions are leading causes of morbidity and mortality worldwide, underscoring the urgent need for effective strategies to prevent or reverse SMD-related disease.

SMD-related diseases commonly co-occur, and the probability of having multiple such conditions increases with age. This pattern suggests that aging disrupts both intraorgan and interorgan signaling networks that normally sustain metabolic resilience, limit tissue degeneration, and support longevity. Dysfunction in a single organ can impose additional demands on other organs, triggering a cascade of compensatory responses that ultimately cause progressive dysfunction in multiple systems. This amplifies SMD, accelerates tissue aging across the body, shortens health span, and reduces lifespan. Although the hallmarks of aging are broadly conserved across tissues, it remains unclear whether there is a final common driver of tissue aging and how cells within a tissue, and tissues within an organism, interact to regulate this process (4). Work in invertebrate models shows that pathways that control energy balance also regulate lifespan. For example, siRNA screens in adult Drosophila have shown that Hedgehog signaling, a lipid-regulated pathway that orchestrates organ development, controls total body energy balance and fat mass (5). Hedgehog pathway activity also regulates lifespan in flies (6, 7). Whether similar coupling between energy balance and aging exists in mammals is unknown. This question is directly relevant to SMD-related diseases, since SMD is characterized by disordered systemic energy homeostasis.

The liver is central to systemic energy regulation and metabolic control. We therefore hypothesized that the liver, and specifically hepatocytes, plays a key role in the development of SMD and related multiorgan dysfunction. Hepatocytes are the primary liver cell type responsible for maintaining systemic metabolic homeostasis. In this study, we asked whether selectively accelerating aging in hepatocytes is sufficient to induce SMD and to accelerate aging in other tissues. If so, we sought to identify the mechanisms that mediate these systemic effects.

We built on our prior work showing that adult hepatocytes rely on developmental morphogen pathways, including Hedgehog, to maintain metabolic flexibility and actively delay liver aging. We previously found that hepatocyte Hedgehog signaling is suppressed in old mice (8). In young mice, conditional deletion of Smoothened (Smo), an essential Hedgehog signaling component, in hepatocytes rapidly induced hepatocyte senescence, impaired liver regeneration (9), and produced other hallmarks of hepatocyte aging within 1 week. Whole-liver transcriptomes from these young mice with Smo-deficient hepatocytes (hereafter, Smo-KO mice) resembled those of old mice (8), demonstrating that loss of hepatocyte Hedgehog signaling accelerates liver biological aging. Consistent with the increased incidence and severity of MASLD with aging in humans (10), young Smo-KO mice developed mild MASLD and SMD (hypertriglyceridemia, disrupted cholesterol homeostasis, and hyperinsulinemia/insulin resistance) on a standard chow diet (11) and rapidly progressed to severe MASLD when fed a choline-deficient high-fat diet (CDA-HFD) that caused only minimal injury in age-matched controls (9). However, the Smo-dependent mechanisms that control liver biological aging are not fully defined, and it is unknown whether hepatocyte-driven liver aging alone is sufficient to accelerate aging in other organs.

Aging is associated with iron accumulation in many tissues that are vulnerable to degeneration in SMD, including the liver (12, 13). In parallel, the healthy liver is the main source of proteins that control both hepatic and systemic iron homeostasis (14). Iron is essential for energy metabolism: it is required for proper function of the electron transport chain and multiple oxidant-generating enzymes, and it modulates metabolism via regulation of hypoxia-responsive factors (15). However, iron bioavailability is tightly controlled because iron can catalyze peroxidation of membrane lipids, and excessive lipid peroxidation can disrupt membranes and cause cell death (16). Failure to restrain iron-driven lipid peroxidation leads to cellular senescence and, ultimately, ferroptosis, an iron-dependent form of regulated cell death. Cells experiencing excessive lipid peroxidation but not yet dead are under “ferroptotic stress” (17, 18). Ferroptotic stress has been implicated in a variety of degenerative diseases characterized by senescent cell accumulation, including MASLD and CKD. We and others have shown that treatment of mouse models of MASLD and CKD with ferrostatin-1 (Fer-1), a ferroptosis inhibitor, reverses tissue damage (19, 20). Notably, several interventions believed to slow biological aging, and which have broad benefit in MASLD and other SMD-related conditions, also inhibit ferroptosis (21). Together, these observations suggest that ferroptosis is a conserved, aging-sensitive process that shapes metabolic stress and tissue aging. Whether Hedgehog signaling regulates ferroptosis to control the rate of biological aging in hepatocytes or in other cell types is unknown.

Since hepatocyte Hedgehog signaling is disrupted in Smo-KO mice, and these mice provide a validated model of hepatocyte-specific senescence, accelerated liver aging, and MASLD, we used them to dissect the roles of hepatocyte Hedgehog signaling, ferroptotic stress, and senescence in SMD-related organ dysfunction. Based on the strong clinical association between MASLD and renal dysfunction (11, 19) and reports that WT mice fed MASLD-inducing diets develop progressive renal injury (22, 23), we also asked whether selectively accelerating hepatocyte aging is sufficient to induce kidney pathology. Our new data provide direct evidence that loss of hepatocyte Hedgehog activity induces ferroptotic stress in hepatocytes, is sufficient to drive hepatocyte senescence, and triggers MASLD and renal damage via dysregulation of vasoactive factors and altered liver perfusion.

## Results

### Human MASLD livers are enriched in Smo-deficient, aging, and ferroptosis-stressed hepatocytes.

Human MASLD livers are known to accumulate senescent hepatocytes (10), and reducing senescent hepatocyte burden improves MASLD in mice (24). Previously, we showed that hepatocytes are the major source of Smo mRNA in healthy human livers and that Smo expression declines as liver injury and fibrosis worsen in MASLD (11). Whether loss of hepatocyte Hedgehog signaling actually drives hepatocyte and liver aging in MASLD has not been clear.

**Figure 1:**
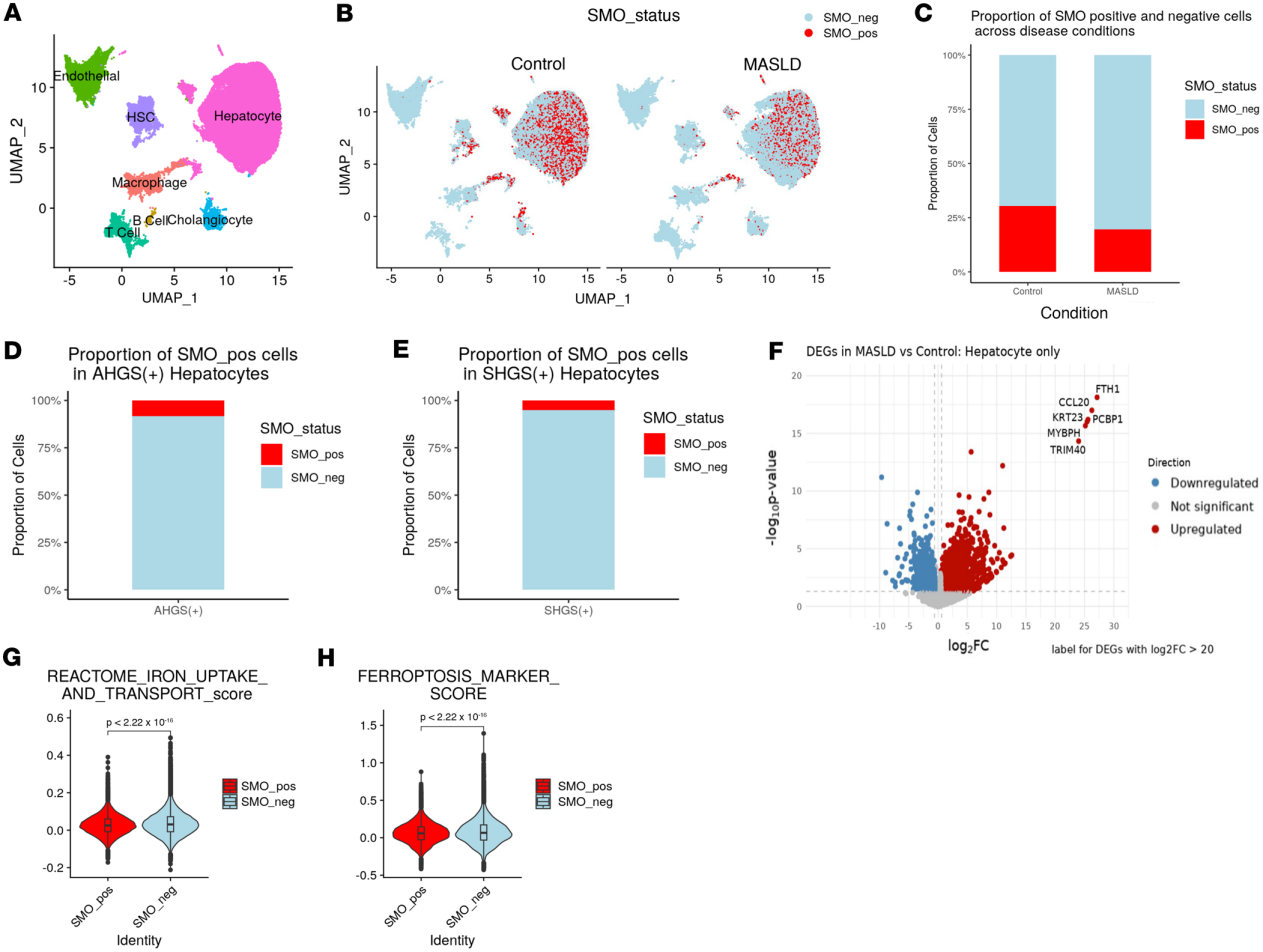
Human MASLD livers are enriched with Smo-deficient hepatocytes that exhibit accelerated aging and ferroptotic stress. (**A**) UMAP showing distinct cell populations identified from single-nucleus sequencing of over 68,000 liver nuclei from 10 individuals. (**B**) UMAP showing cells in control and MASLD samples classified as either SMO_pos (expressing detectable SMO transcripts) or SMO_neg (lacking detectable SMO transcripts). (**C**) Proportion of SMO_pos and SMO_neg cells in control and MASLD liver. (**D** and **E**) Proportion of SMO_pos and SMO_neg cells in AHGS-positive or SHGS-positive hepatocytes. (**F**) Volcano plot showing FTH1 was the most upregulated transcript in patients with MASLD. (**G** and **H**) Violin plots showing enrichment for gene signature indicative of iron accumulation and ferroptosis. *P* value was calculated using Wilcoxon’s rank-sum test in **G** and **H**.

To address this, we analyzed single-nucleus RNA-Seq data from human liver samples from a Duke cohort, using gene signatures that distinguish old from young hepatocytes (aging hepatocyte gene signature, AHGS) and senescent from nonsenescent hepatocytes (senescent hepatocyte gene signature, SHGS) (25). We first used uniform manifold approximation and projection (UMAP) visualization to identify hepatocytes among all liver cell types in control and MASLD samples (Figure 1A). We then subdivided hepatocytes into Smo(+) and Smo(–) populations based on detectable Smo transcripts (Figure 1B). Hepatocytes from MASLD livers (*n =* 5) were relatively depleted of Smo(+) cells compared with hepatocytes from age-matched non-MASLD controls (*n =* 5) (Figure 1C). Importantly, hepatocyte subpopulations marked by aging (AHGS) or senescence (SHGS) signatures were almost exclusively Smo(–) (Figure 1, D and E). FTH1 (the gene that encodes ferritin heavy chain) was the most upregulated transcript in hepatocytes of our MASLD cohort (Figure 1 F). Consistent with this finding, Smo(–) hepatocytes in MASLD livers were enriched for gene signatures indicating iron accumulation and ferroptosis (Figure 1, G and H). These data suggest that in human MASLD, hepatocytes that lack Smo, and thus have impaired Hedgehog signaling, are preferentially enriched among aging, senescent, and ferroptosis-stressed hepatocytes.

### Hepatocyte-specific Smo deletion promotes ferroptotic vulnerability and disturbs hepatic and systemic iron homeostasis.

Our earlier work showed that Smo deletion in mouse hepatocytes causes hepatic steatosis (11). Other studies indicate that excessive hepatic iron promotes triglyceride accumulation in hepatocytes (19) and increases the risk of ferroptosis by enhancing iron-driven lipid peroxidation (19). Aging itself increases iron content in many tissues, including the liver (26). We therefore asked whether Smo deletion increases liver iron.

**Figure 2:**
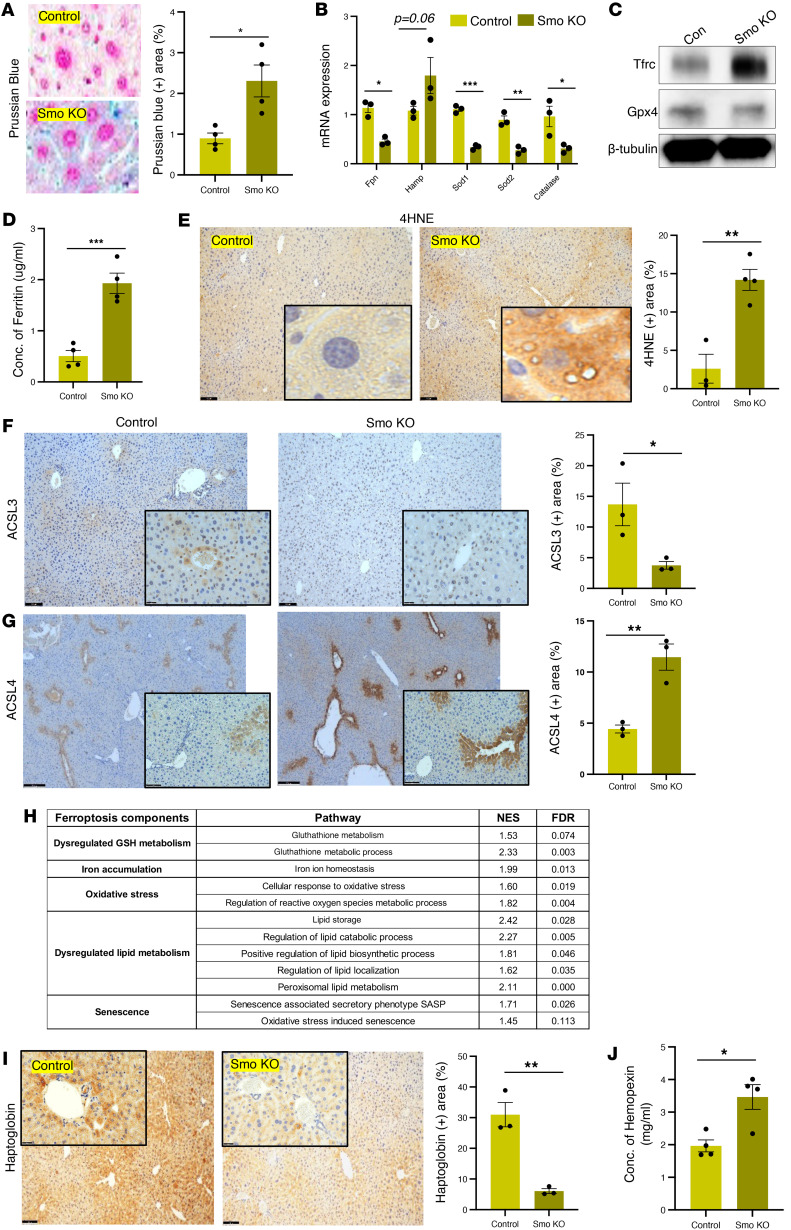
Hepatocyte-specific deletion of Smo induces hepatic iron accumulation and ferroptotic stress. (**A**) Prussian blue staining showing increased hepatic iron in Smo-KO mice versus controls. Scale bar: 50 μm (upper panels). (**B**) qRT-PCR of iron regulatory genes (Fpn, Hamp) and oxidative stress markers (Sod1, Sod2, catalase); *n* = 3/group. (**C**) Representative immunoblot of iron transporter (Tfrc) and glutathione peroxidase 3 (Gpx4) in Smo-KO compared with control mice. β-tubulin was loading control. (**D**) Serum ELISA of ferritin levels in Smo-KO versus control mice (*n* = 4 mice/group). (**E**–**G**) IHC of 4-hydroxynonenal (4-HNE), ACSL3, ACSL4 in Smo-KO mice compared with controls. Scale bars: 100 μm; insets, 20 μm. Quantitative data shown alongside images. (**H**) Gene ontology (GO) analysis showing enrichment of pathways related to dysregulated glutathione metabolism, iron homeostasis, oxidative stress, and lipid metabolism in hepatocytes from Smo-KO mice. (**I**) IHC of haptoglobin in Smo-KO mice compared with controls. Scale bars: 100 μm; inset, 20 μm. (**J**) Serum ELISA of hemopexin levels in Smo-KO mice. Data represent mean ± SEM; statistical significance was determined using unpaired 2-tailed *t* test; *P ≤ 0.05; **P ≤ 0.01; ***P ≤ 0.001.

We stained liver sections from young Smo-KO mice and age-matched Smo^fl/fl^ controls with Prussian blue to detect iron. Smo deletion increased hepatic iron deposition (Figure 2A). Cellular iron levels are normally tightly controlled: transferrin-bound iron enters cells via transferrin receptor (Tfrc), and iron is exported by ferroportin (Fpn). Hepcidin (Hamp) inhibits iron export by destabilizing Fpn. Compared with controls, hepatocytes from Smo-KO mice showed decreased Fpn expression and increased Tfrc and Hamp expression (Figure 2, B and C), indicating increased iron import and reduced iron export.

As iron stores rise, cells typically upregulate ferritin (FTH1) to sequester iron and limit its reactive pool. Ferritin expression was increased in Smo-KO hepatocytes (Figure 2C). Because the liver is the principal source of circulating ferritin, serum ferritin levels were approximately 4-fold higher in Smo-KO mice than in controls (Figure 2D). Despite this compensatory ferritin induction, Smo-KO hepatocytes accumulated more 4-hydroxynonenal (4-HNE), a marker of lipid peroxidation, than control hepatocytes (Figure 2E), indicating that labile iron remained sufficient to drive lipotoxicity.

Ferroptosis susceptibility is also shaped by the composition of membrane lipids. ACSL4 promotes incorporation of polyunsaturated fatty acids (PUFAs) into phospholipids, generating substrates for peroxidation during ferroptosis. In contrast, ACSL3 favors monounsaturated fatty acids (MUFAs), which are less prone to peroxidation and can protect against ferroptosis. In Smo-KO livers, ACSL4 expression was increased in hepatocytes and upregulated relative to ACSL3, whereas control livers showed the opposite pattern (Figure 2, F and G). This suggests that Smo-KO hepatocytes are enriched in PUFA-containing membrane lipids, increasing their susceptibility to peroxidation.

Antioxidant defenses were also impaired. Enzymes that detoxify superoxide and hydrogen peroxide (e.g., superoxide dismutase and catalase) were reduced in Smo-KO hepatocytes (Figure 2B). As noted above, GPx4, the key enzyme that detoxifies lipid peroxides, was also decreased (Figure 2C), further favoring lipid radical accumulation. Gene set enrichment analysis (GSEA) of bulk RNA-Seq data from Smo-KO versus control hepatocytes showed dysregulation of pathways involved in glutathione metabolism, iron handling, oxidative stress responses, and lipid metabolism (Figure 2H).

To confirm these findings at single-cell resolution, we isolated primary hepatocytes from an independent set of young Smo-KO and control mice and performed single-cell RNA-Seq analysis on approximately 10,000 hepatocytes. UMAP showed that most cells in both groups expressed HNF4α (a mature hepatocyte marker), but Smo-deleted hepatocytes (Cre-treated, blue) clustered separately from control hepatocytes (luciferase treated, pink) (Supplemental Figure 1, A and B; supplemental material available online with this article; https://doi org/10.1172/jci.insight.207066DS1), confirming broad transcriptomic reprogramming after Smo deletion. Within these clusters, Smo-deleted hepatocytes were enriched for genes promoting iron uptake (TFRC, Slc11a2, Steap3, HJV) (Figure 1C), and for an independently defined ferroptosis-driver gene signature (27) (Supplemental Figure 1D).

Thus, in Smo-KO mice, disruption of hepatocyte Hedgehog signaling is sufficient to increase hepatic iron accumulation and to shift hepatocyte lipid and redox pathways toward ferroptotic stress. These findings mirror the changes seen in Smo(–) human hepatocytes (Figure 1, F and G) and, together with prior work showing that Smo deletion disrupts other key ferroptosis determinants such as lipid metabolism and oxidant balance (9, 28), explain how Smo loss induces ferroptotic stress in young hepatocytes. We have also shown that ferroptotic stress is markedly increased in hepatocytes of old mice (19). Hence, these data further support the idea that Smo normally acts to restrain hepatocyte biological aging. Notably, staining for macrophages (F4/80) was similar in livers of Smo-KO and control mice (data not shown), consistent with previous evidence that this level and duration of metabolic stress do not yet produce overt steatohepatitis.

Systemic iron homeostasis is largely governed by 4 liver-derived serum proteins: transferrin, hepcidin, haptoglobin, and hemopexin. Transferrin binds iron and delivers it to cells via transferrin receptors. Hepcidin limits systemic iron exposure by blocking iron export from enterocytes and retaining hemoglobin-derived iron within macrophages. Haptoglobin and hemopexin bind free hemoglobin and free heme, respectively, preventing these iron-containing molecules from undergoing auto-oxidation and releasing ferrous iron and ROS (29). In haptoglobin deficiency, hemopexin levels often increase to compensate, but if this is inadequate, the kidney is exposed to excess free heme (30).

In Smo-KO mice, hepatic transferrin expression was similar to controls (data not shown), but hepatic haptoglobin expression was decreased, and serum hemopexin levels were increased (Figure 2, I and J). These changes suggest impaired iron-scavenging capacity and potential sensitization of peripheral organs, such as the kidney, to iron-mediated oxidative stress.

### Hepatocyte-specific Smo deletion induces renal senescence via altered systemic iron regulation.

We next asked whether the changes in liver-derived iron-regulatory proteins induced by hepatocyte-specific Smo deletion affected the kidney. We compared kidneys from chow-fed Smo-KO and control mice after confirming that Cre-mediated recombination effectively depleted Smo in hepatocytes (Supplemental Figure 2A) but did not alter Smo expression in the kidney (Supplemental Figure 2B).

**Figure 3:**
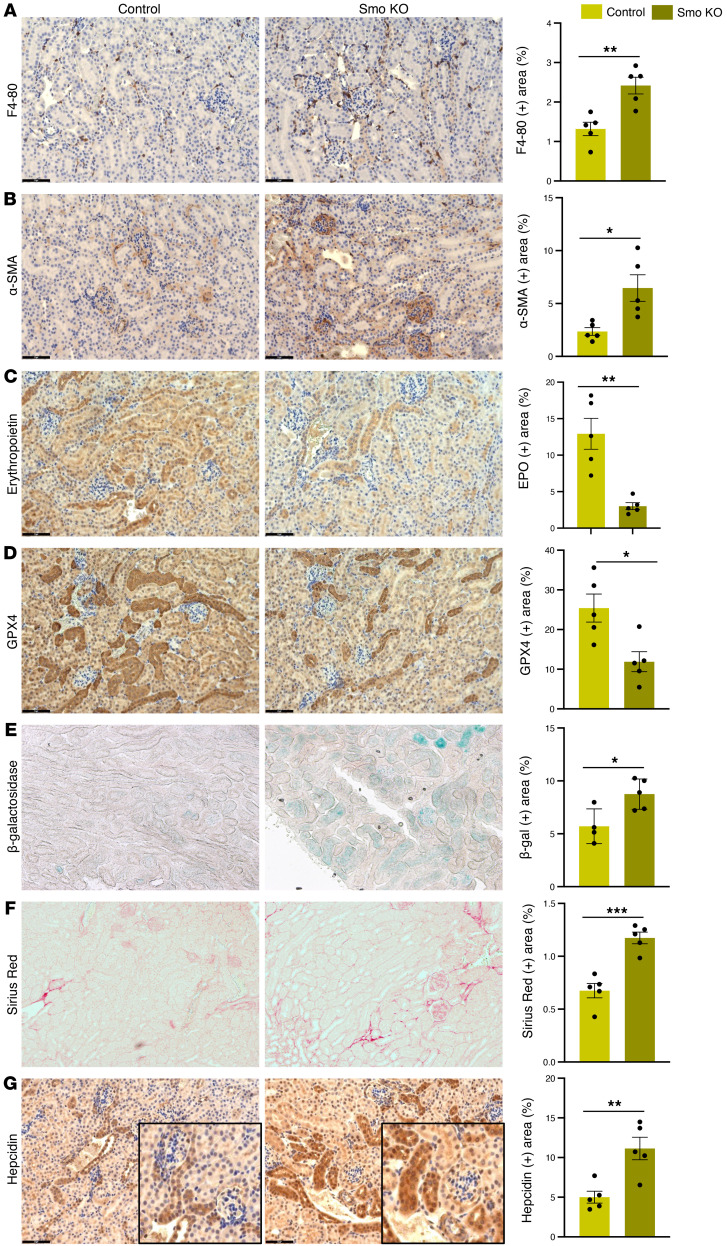
Hepatocyte-specific Smo deletion induces renal ferroptotic stress, senescence, inflammation, and fibrosis. (**A**–**D**) IHC of F4/80, α-smooth muscle actin (α-SMA), erythropoietin, and GPX4 in kidney of Smo-KO mice versus control. (**E** and **F**) β-gal and Sirius red staining in Smo-KO mice versus control kidney. (**G**) IHC of hepcidin in kidney of Smo-KO mice versus control. Quantification shown adjacent to respective panels. Scale bars: 100 μm; insets: 20 μm. Data represent mean ± SEM; statistical significance was determined using unpaired 2-tailed *t* test; *P ≤ 0.05; **P ≤ 0.01; ***P ≤ 0.001.

Compared with normal-appearing control kidneys, kidneys from Smo-KO mice showed increased accumulation of macrophages and myofibroblasts and reduced expression of erythropoietin (Epo), a marker of healthy renal tubular cells (RTCs) (Figure 3, A–C) (31). GPx4 expression was significantly reduced in RTCs, and senescence-associated β-galactosidase staining was increased (Figure 3, D and E). Interstitial collagen deposition was also significantly higher (Figure 3F). Although Prussian blue staining did not reveal clear increases in iron within RTCs (data not shown), these cells showed strong hepcidin immunoreactivity (Figure 3G). Circulating liver-derived hepcidin is filtered into the urine and reabsorbed by RTCs, where it locally inhibits Fpn and promotes iron retention (32). Excess iron can inhibit Epo transcription through mechanisms involving increased renal oxidative stress (33).

Overall, these data indicate that inducing ferroptotic stress in hepatocytes by deleting Smo is sufficient not only to cause hepatic steatosis but also to provoke secondary kidney injury characterized by increased RTC hepcidin, reduced RTC GPx4 and Epo, tubular cell senescence, macrophage and myofibroblast accumulation, and interstitial fibrosis. Strikingly, although the primary insult was confined to hepatocytes, macrophage accumulation and fibrosis were more pronounced in the kidneys than in the liver.

### Ferroptotic stress makes steatotic hepatocytes more vulnerable to secondary hits, accelerating transition to steatohepatitis, fibrosis, and renal injury.

“Simple steatosis” is often considered relatively benign clinically, but it is thought to increase susceptibility to additional metabolic or toxic stressors (“second hits”), thereby lowering the threshold for progression to metabolic dysfunction–associated steatohepatitis (MASH) and maladaptive fibrogenic repair (34). The mechanisms by which steatosis increases vulnerability to MASH, and why progression rates vary widely between and within individuals, are not fully understood. Older age is a recognized risk factor for both liver disease progression and development of MASH-associated comorbidities (35).

**Figure 4:**
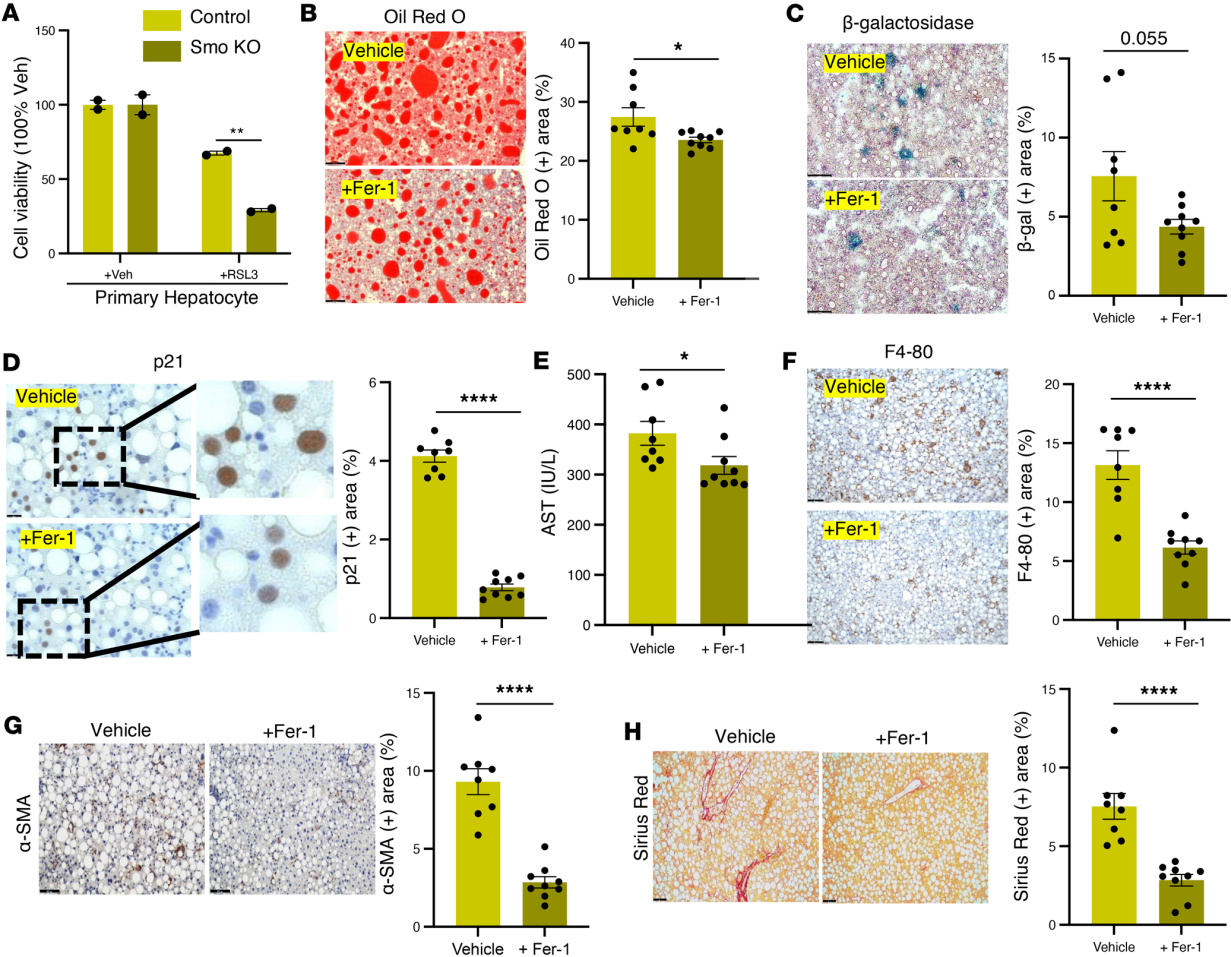
Ferrostatin-1 treatment alleviates hepatic ferroptotic stress and attenuates MASH progression in Smo-KO mice. (**A**) Cell viability of primary hepatocytes isolated from Smo-KO and control mice after GPx4 inhibitor RSL3 treatment. (**B**) Oil Red O staining of liver after Fer-1 administration during CDA-HFD feeding. (**C**) β-galactosidase staining of liver in vehicle or Fer-1–treated Smo-KO mice. (**D**) IHC of p21 in vehicle or Fer-1–treated Smo-KO mice. (**E**) Serum AST in vehicle or Fer-1–treated mice. (**F** and **G**) F4/80 and α-SMA IHC in livers of vehicle or Fer-1–treated Smo-KO mice. (**H**) Sirius red staining of liver in vehicle or Fer-1–treated Smo-KO mice. Quantitative data are shown adjacent to corresponding images. Scale bars: 100 μm. Data represent mean ± SEM; statistical significance was determined using unpaired 2-tailed *t* test; *P ≤ 0.05; **P ≤ 0.01; ****P ≤ 0.0001.

We previously reported that, similar to very old WT mice, young Smo-KO mice develop substantially more severe MASH and liver fibrosis than age-matched controls when exposed briefly to MASH-inducing diets (9). To test the role of ferroptotic stress in determining hepatocyte susceptibility to secondary insults, we isolated primary hepatocytes from Smo-KO and control mice and assessed their viability before and after exposure to RSL3, a GPx4 inhibitor that induces ferroptosis. At baseline, despite increased steatosis and oxidized lipid accumulation, Smo-KO hepatocyte viability was similar to controls. However, upon RSL3 treatment, Smo-KO hepatocytes were significantly more susceptible to ferroptosis (Figure 4A).

To determine whether ferroptotic stress in these viable but vulnerable Smo-depleted hepatocytes promotes the progression from steatosis to steatohepatitis and contributes to renal dysfunction in vivo, we fed young Smo-KO mice a CDA-HFD to induce MASH, treated them with Fer-1 or vehicle during the final week, and examined whether ferroptosis inhibition could prevent or reverse disease progression.

Fer-1 treatment reduced hepatic Oil Red O staining (steatosis) and senescence-associated β-galactosidase activity (Figure 4, B and C). Nuclear accumulation of the cell-cycle inhibitor p21 in hepatocytes and serum AST levels, a marker of liver injury, also decreased (Figure 4, D and E). In addition, macrophage and myofibroblast accumulation was reduced (Figure 4, F and G), and fibrosis, assessed by Sirius red staining, was significantly attenuated (Figure 4H). These data support the concept that increased ferroptotic stress in Smo-depleted hepatocytes lowers the threshold for transition from steatosis to MASH, thereby accelerating MASLD progression and fibrosis risk in Smo-KO mice.

### Inhibiting hepatic ferroptosis improves renal injury and dysfunction in metabolic steatohepatitis.

**Figure 5:**
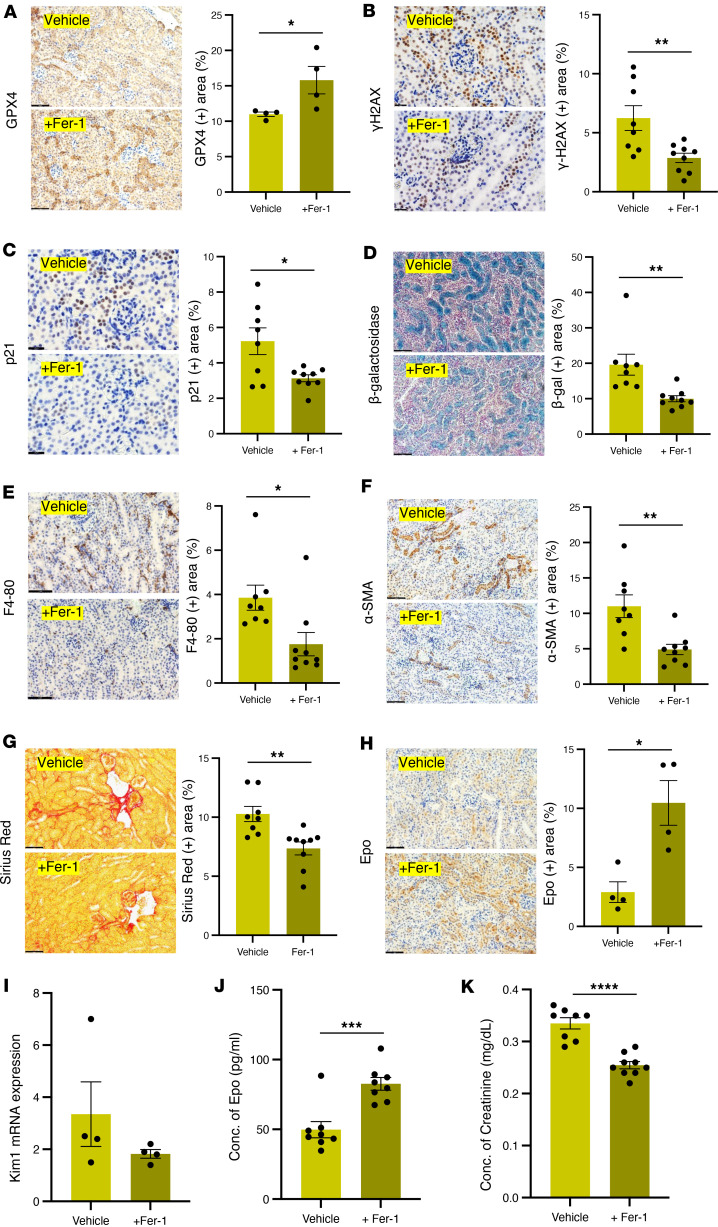
Ferrostatin-1 treatment mitigates renal ferroptotic stress, inflammation, fibrosis, and dysfunction in Smo-KO mice fed CDA-HFD. (**A**) GPx4 immunostaining in renal tubular cells of Fer-1–treated mice compared with vehicle controls. (**B**–**D**) γH2AX (**B**) and p21 (**C**) immunostaining, and β-galactosidase staining (**D**), in kidneys of Fer-1–treated mice. (**E** and **F**) IHC for F4/80 and α-SMA in Fer-1–treated kidneys. (**G**) Sirius red staining after Fer-1 treatment. (**I**) Kim1 expression in Fer-1–treated mice. Renal erythropoietin (Epo) expression (**H**) and circulating Epo levels (**J**) after Fer-1 treatment. (**K**) Serum creatinine levels in Fer-1–treated mice. Quantitative data are presented adjacent to each image. Scale bars: 100 μm. Data are shown as mean ± SEM; statistical significance was determined using unpaired 2-tailed *t* test; *P ≤ 0.05; **P ≤ 0.01; ***P ≤ 0.001; ****P ≤ 0.0001.

Remarkably, Fer-1 also improved kidney injury and dysfunction in CDA-HFD–fed Smo-KO mice. Compared with vehicle-treated Smo-KO controls, kidneys from Fer-1–treated Smo-KO mice displayed increased GPx4 expression and reduced γ-H2AX (a marker of DNA damage), p21, and senescence-associated β-galactosidase activity (Figure 5, A–D). These changes indicate reduced renal oxidative stress and improved regenerative capacity. Macrophage and myofibroblast accumulation decreased, and renal fibrosis was reduced (Figure 5, E–G).

Functional indices also improved. Renal Kim-1 expression, a marker of tubular injury, declined (Figure 5I), while renal Epo expression and serum Epo levels increased (Figure 5, H and J). Serum creatinine, a marker of renal failure, decreased (Figure 5K). Collectively, these findings show that ferroptotic stress in hepatocytes of steatotic livers is sufficient to induce kidney stress and damage, and that inhibiting ferroptosis can ameliorate renal injury and dysfunction, as well as MASH. This may explain the strong clinical association between progressive steatotic liver disease and CKD in patients with MASH (36).

### Vascular dysfunction and altered iron homeostasis precede overt liver failure in patients with MASH.

The liver and kidney coordinate iron homeostasis through mechanisms that also affect erythropoiesis and vascular function. As noted, hepatocytes produce hepcidin (Hamp), which reduces systemic iron availability by limiting intestinal iron absorption and iron release from macrophages. The kidney contributes by filtering iron and hepcidin at the glomerulus and reabsorbing them in renal tubules, enabling their recycling. Some RTCs also synthesize hepcidin locally, which limits renal iron export, preserves iron for renal metabolism, but increases vulnerability to ferroptosis (37). Iron further regulates renal Epo production, and kidney-derived Epo suppresses hepatic hepcidin expression to ensure adequate systemic iron for erythropoiesis (38). EPO also promotes hepatocyte survival (39) and protects rodents against diet-induced obesity, steatohepatitis, insulin resistance, and renal inflammation (40, 41). In addition, EPO enhances VEGF-α production, supporting hepatic vasculogenesis (42, 43).

The renin-angiotensin system (RAS) links liver and kidney function at the vascular level. Hepatocyte-derived angiotensinogen (Agt) is converted to angiotensin II (AT2) by renin, a kidney-derived enzyme. Beyond regulating blood pressure and flow, AT2 modulates renal expression of iron-regulatory factors, including Hamp and Fpn (44). Dysregulated RAS activity is believed to underlie systemic circulatory dysfunction, renal failure, and multiorgan injury in advanced liver disease, as the liver depends on stable perfusion gradients of oxygen, hormones, and substrates to maintain energy homeostasis. Clinically, blood-based scores predicting hepatic decompensation and multiorgan failure incorporate creatinine as an indicator of renal dysfunction (45). Within this context, our Smo-KO mouse data suggest that disruption of hepatocyte Hedgehog signaling may contribute to both liver and kidney dysfunction by altering expression of iron-sensitive mediators that interact with the RAS.

**Figure 6:**
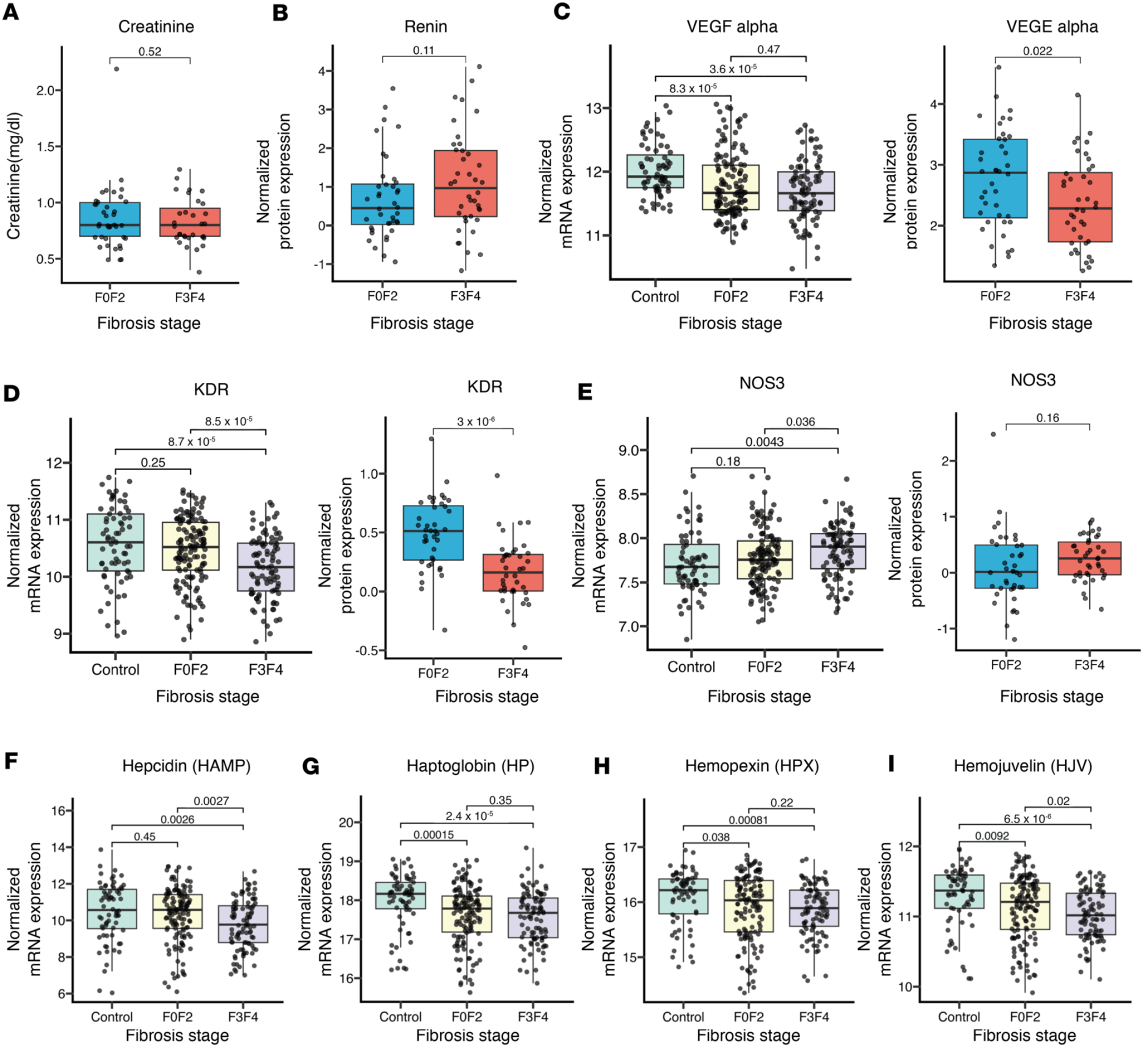
Circulatory dysfunction and altered iron regulatory proteins are associated with advanced liver fibrosis in patients with MASH. Box plots showing comparative serum protein levels and liver mRNA expression in patients with MASH grouped by fibrosis stage (F0–F2 vs. F3–F4) and healthy controls. (**A** and **B**) Serum creatinine and renin levels in advanced fibrosis (F3–F4) versus control (**C** and **D**) serum VEGFA, liver VEGFA mRNA, and serum KDR (VEGF receptor) levels. (**E**) Serum and liver expression of NOS3. (**F**–**I**) Liver expression of hepcidin, haptoglobin, hemopexin, and hemojuvelin. *P* values are indicated on each panel; data points represent individual patients. *P* value was calculated using Wilcoxon’s rank-sum test.

Because Smo(+) hepatocytes are reduced in human MASLD, we examined O-link proteomic data from a clinical cohort of patients with early (fibrosis stage F0–2) versus advanced (F3–4) MASH to assess markers of circulatory dysfunction. Clinical and histological features of this cohort have been reported previously (46, 47). Serum creatinine levels were similar between early and advanced fibrosis groups (Figure 6A), but serum renin levels were modestly higher in advanced fibrosis (Figure 6B). Both hepatic mRNA and serum protein levels of VEGF-α and its receptor VEGFR2/KDR were decreased in advanced versus early fibrosis (Figure 6, C and D), whereas hepatic NOS3 (endothelial NOS) expression and serum NOS3 levels were increased (Figure 6E). These findings show that alterations in vasogenic and vasoactive factors from the liver and kidney accompany fibrosis progression and occur before overt liver failure or renal dysfunction.

In parallel, we observed altered expression of several iron regulatory factors (hepcidin, haptoglobin, hemopexin, hemojuvelin) at the liver transcript and/or serum protein level in this cohort (Figure 6, F–I), indicating systemic disruption of iron homeostasis. These findings are consistent with prior reports that 30% or more of patients with MASLD have systemic iron overload, termed dysmetabolic iron overload syndrome, which is associated with more severe liver damage and worse cardiovascular comorbidities (48). However, patients with MASH are heterogeneous with respect to comorbidities and treatments, limiting the ability of researchers to isolate the specific role of hepatocytes in iron loading, vascular pathology, and end-organ damage.

### Ferroptosis inhibition restores hepatic perfusion by reversing hepatocyte-induced vascular dysfunction in experimental MASLD.

**Figure 7:**
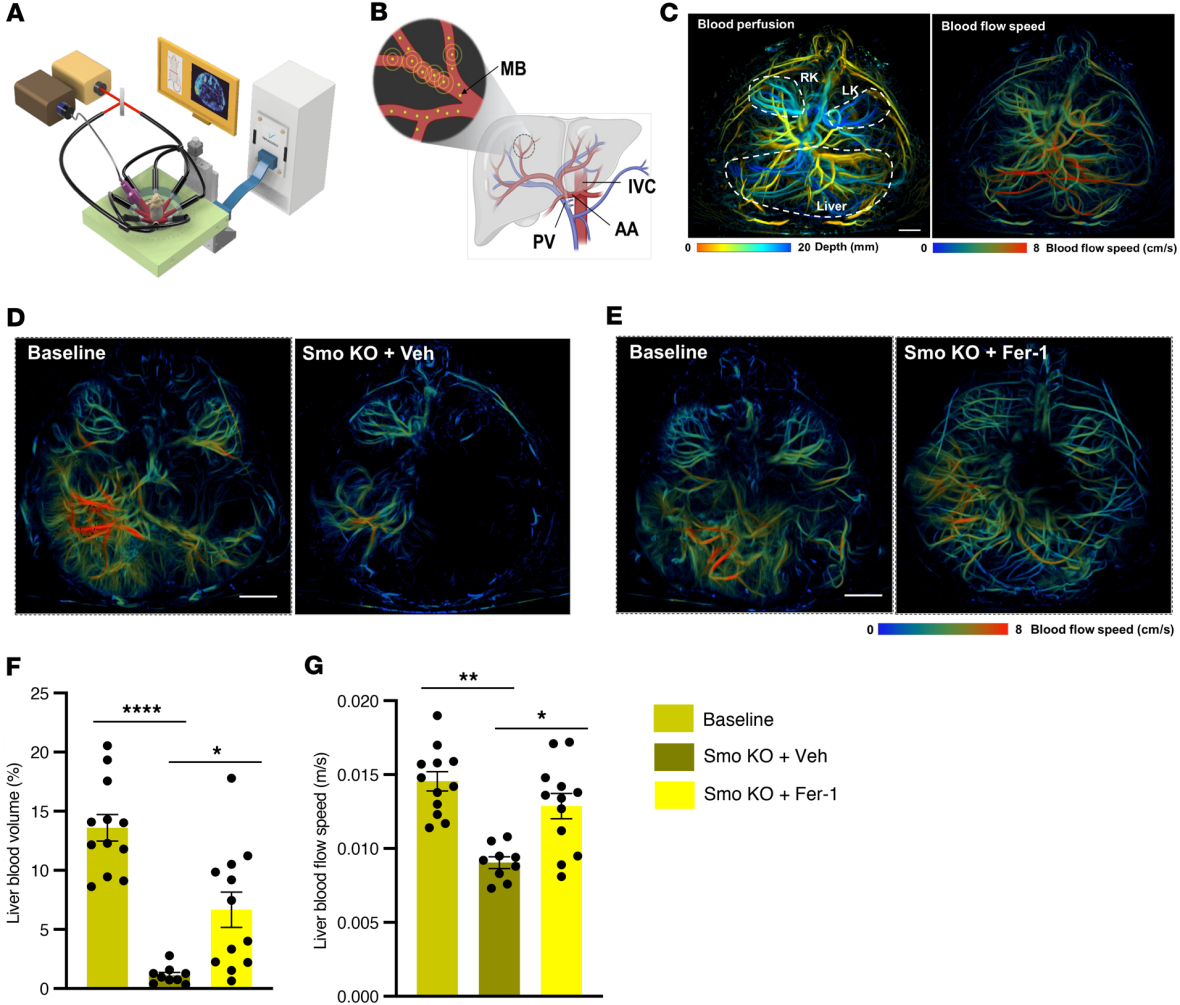
Ferrostatin-1 restores hepatic blood perfusion and reverses vascular dysfunction induced by hepatocyte ferroptotic stress in Smo-KO mice. (**A**) Schematic diagram of ultrasound localization microscopy (ULM) for whole-body perfusion imaging. (**B**) The imaging principle of ULM, tracking the gas-filled microbubbles flowing in the blood vessels of the liver. MB, microbubbles; IVC, inferior vena cava; PV, portal vein; AA, abdominal aorta. (**C**) Representative ULM images of the mouse liver and kidney regions, showing the super-resolution vascular image and the blood flow map. LK, left kidney; RK, right kidney. (**D** and **E**) Representative in vivo images of the blood perfusion (**D**) and blood flow speed (**E**) in the liver and kidney at the baseline and after treatment, showing reduced blood perfusion after CDA-HFD in the Smo-KO mice. Scale bars: 1 mm. (**F** and **G**) Statistical analysis of the liver blood perfusion (**F**) and blood flow speed (**G**) at the baseline and with treatments (*n* = 4 mice per group, 4−5 regions of interest per mouse). Data represent mean ± SEM; statistical significance was determined using 2-way ANOVA; *P ≤ 0.05; **P ≤ 0.01; ****P ≤ 0.0001.

To directly examine the link between hepatocyte ferroptotic stress, liver perfusion, and kidney blood flow, we used noninvasive ultrasound localization microscopy (ULM) to measure hepatic and renal blood flow in living CDA-HFD–fed control mice, Smo-KO mice, and Smo-KO mice treated with Fer-1 (Figure 7A; Supplemental Figure 3). ULM tracks gas-filled microbubbles in the circulation to quantify organ perfusion and vessel-level flow velocities (Figure 7B and Supplemental Figure 4) (49). This technique allows high-resolution, longitudinal assessment of organ blood flow over 6 weeks without invasive procedures (Figure 7C and Supplemental Figure 5 and 6).

Longitudinal imaging demonstrated that CDA-HFD significantly reduced liver blood volume (perfusion) and flow speed in Smo-KO mice (Figure 7, D, F, and G, and Supplemental Figure 7). Renal perfusion was somewhat decreased (Figure 7D), though not statistically significant (data not shown and Supplemental Figure 8). Strikingly, Fer-1 treatment restored hepatic blood flow and perfusion in Smo-KO mice (Figure 7E; Figure 7, F and G). Taken together, these data show that Hedgehog signaling regulates blood flow by determining ferroptotic stress levels in hepatocytes.

**Figure 8:**
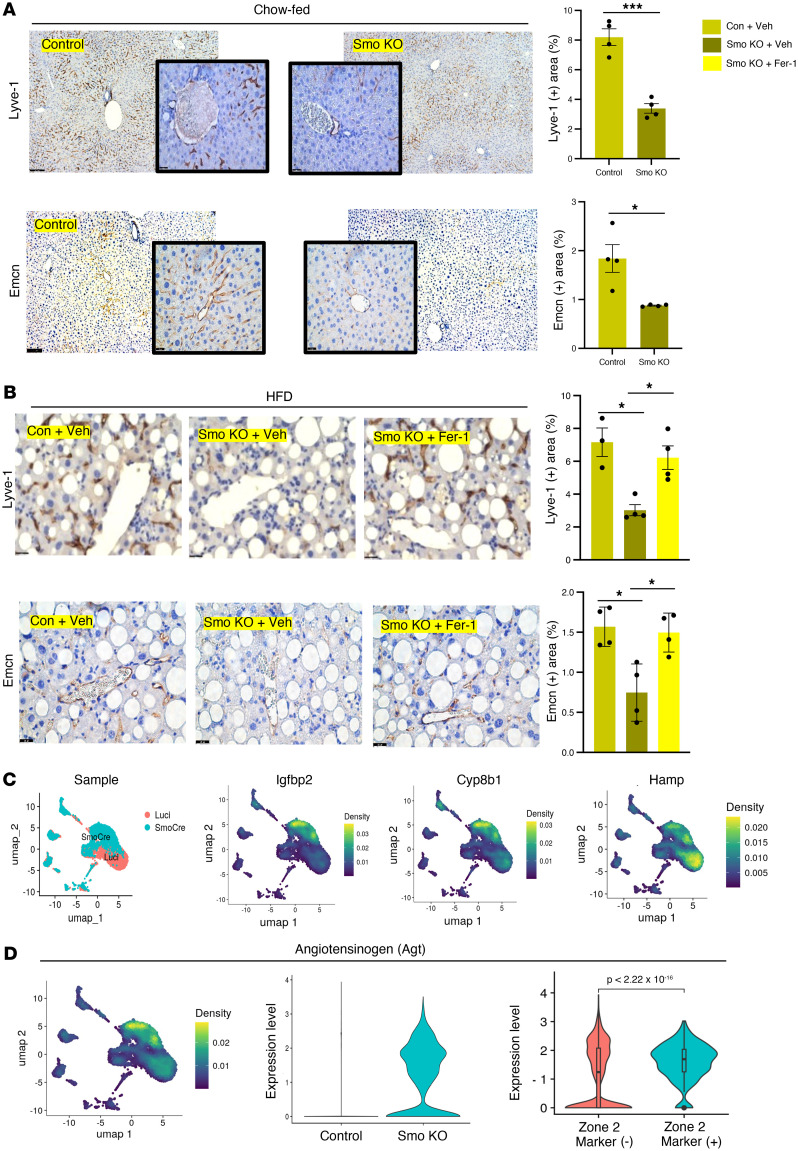
Ferrostatin-1 attenuates liver sinusoidal function and zonation in Smo-KO mice. (**A** and **B**) IHC staining of hepatic sinusoidal endothelial marker, Lyve-1 and Emcn (Endomucin) in chow-fed (**A**) and CDA-HFD–fed (**B**) Smo-KO mice. (**C**) scRNA-Seq results showing expression density of Igfbp2, Cyp8b1, Hamp, and angiotensinogen (Agt) in control and Smo-KO hepatocytes. (**D**) Violin plots showing Agt expression level in control and Smo-KO hepatocytes, and in zone 2 marker–positive versus –negative hepatocytes. Statistical significance was determined using unpaired 2-tailed *t* test; *P ≤ 0.05; ***P ≤ 0.001.

To investigate mechanisms linking hepatocyte ferroptotic stress to sinusoidal dysfunction, we examined liver sinusoidal endothelial cell (LSEC) markers by IHC. In chow-fed controls, the LSEC marker Lyve-1 was most prominently expressed in mid-zonal (zone 2) sinusoids. In contrast, livers from Smo-KO mice showed reduced Lyve-1 staining consistent with sinusoidal capillarization (Figure 7A), whereas Fer-1 treatment restored Lyve-1 expression (Figure 7B). Iron retention in the microenvironment has been reported to suppress Lyve-1 in other tissues (50). Another LSEC marker, endomucin 2 (Emcn2), showed similar changes with Smo-KO and Fer-1 treatment (Figure 8, A and B). Loss of Emcn2 is expected to impair VEGF/VEGFR2 signaling, which is needed for proper angiogenesis (51).

Single-cell transcriptomic analysis of control and Smo-KO hepatocytes revealed that Smo(–) hepatocytes were relatively depleted of mRNAs encoding EpoR, VEGF-α, and VEGFR2/KDR (Supplemental Figure 1D). This is notable because EPO-EpoR signaling has been shown to activate Sonic Hedgehog/Smo signaling and induce VEGF-α transcription in other cell types (52), and VEGF-α/VEGFR2 signaling supports hepatocyte survival in injured livers (53). Smo(–) hepatocyte clusters that were enriched for iron-regulatory genes and ferroptosis-driver signatures (Supplemental Figure 1, A–C) were also enriched for zone 2 markers (e.g., Igfbp2, Cyp8b1, Hamp), suggesting spatial colocalization of hepatocyte ferroptotic stress and sinusoidal dysfunction.

Remarkably, angiotensinogen (Agt), a key RAS component and regulator of sinusoidal blood flow, was also highly expressed in zone 2 hepatocytes and more abundant in Smo-deleted hepatocytes than in controls (Figure 8, C and D). These findings suggest that vasoactive factors produced by metabolically stressed, senescing hepatocytes can actively remodel intrahepatic blood flow. Thus, this preclinical model identifies Hedgehog-deficient, metabolically stressed, senescent hepatocytes as initiators of vascular dysfunction and multiorgan injury in MASLD, and supports further work to identify the specific mediators and downstream targets.

## Discussion

The links between aging, metabolic dysfunction, and multiorgan pathology remain poorly defined. We previously showed that hepatocyte Hedgehog activity is reduced in old mice and that hepatocyte-specific Smo deletion in young mice models accelerated liver biological aging (8, 11). In the current study, we used this model to investigate how aging-sensitive mechanisms in hepatocytes influence metabolic resilience and the health of both the liver and the kidney, an organ that often becomes dysfunctional in patients with advanced liver disease (54).

Aging is associated with increased iron accumulation (55), and here we show that Smo-deficient hepatocytes are at increased risk of ferroptosis, an iron-dependent, regulated cell death driven by lipid peroxidation. We provide evidence that Smo deletion disrupts hepatocyte iron homeostasis in addition to impairing lipid metabolism (8, 28) and increasing oxidative stress (9). Crucially, we demonstrate that the degree of ferroptotic stress in hepatocytes is a key determinant of liver susceptibility to biological aging, as pharmacological inhibition of ferroptosis eliminates the MASLD-susceptible phenotype in young mice with hepatocyte-specific Smo deletion.

These murine findings have direct clinical relevance. Another group recently reported enhanced ferroptotic stress in primary human hepatocytes, and human iPSC-derived hepatocytes, from donors with the PNPLA3 Il128M polymorphism that is an acknowledged risk factor for cirrhosis and liver cancer (56). Hepatocytes with PNPLA3Il128M exhibited membrane enrichment with polyunsaturated lipids and mitochondrial dysfunction, factors that promote susceptibility to ferroptosis, and treatments that inhibited ferroptosis restored their viability. An independent report from Nonalcoholic Steatohepatitis (NASH) Clinical Research Network investigators recently demonstrated that the harmful effects of this PNPLA3 polymorphism on major adverse liver outcomes is markedly increased by older age in humans (57). Previously, we reported that deleting Smo to disrupt hepatocyte Hedgehog signaling in young mice accelerates aging (11). Here, we show that Smo(+) hepatocytes are shielded from ferroptotic stress and senescence but become relatively depleted in human MASLD, and that hepatocyte populations bearing aging or senescence signatures are composed largely of Smo(–) cells. This is, to our knowledge, the first demonstration that aging and senescent hepatocyte populations in human MASLD are predominantly Smo-deficient, and thus prone to ferroptosis. These results have important therapeutic implications, because interventions that lower senescent hepatocyte burden improve liver aging, MASH, and fibrosis in rodent models (9, 25).

We are beginning to understand how disabling Hedgehog signaling and increasing ferroptotic stress in hepatocytes accelerates aging. DNA damage from chronic ferroptotic stress drives hepatocytes into terminal growth arrest (senescence). During this transition, senescent hepatocytes adopt a series of senescence-associated secretory phenotypes (SASPs) that regulate their own survival and generate complex secretomes that reshape the microenvironment and orchestrate wound-healing responses (9). Our human and mouse single-cell data suggest that this biology is particularly robust in zone 2 hepatocytes.

Prior work has shown that zone 2 hepatocytes have stem/progenitor-like properties and are characterized by strong expression of Hamp and Cyp8b1 (58), which regulate iron and cholesterol homeostasis. Proper handling of iron (59) and cholesterol (60, 61) is critical for maintaining stem and progenitor cell pools in many tissues. Stem cell exhaustion is a hallmark of aging and arises as stem cells become senescent, while their SASPs remodel the niche in ways that further impair stem cell function, leading to disordered tissue architecture and eventual organ failure (62). The mechanisms that enable zone 2 hepatocytes to maintain stem/progenitor-like functions in the adult liver remain incompletely understood.

Our current data provide evidence that hepatocyte Hedgehog signaling controls this “stemness” capacity. Previously, we showed that hepatocyte Smo activity regulates hepatic and systemic cholesterol homeostasis (28). Here, we add that hepatocyte Smo also coordinates expression of multiple genes governing local and systemic iron availability. Although the detailed molecular mechanisms remain to be fully worked out, this coupling of Hedgehog signaling to iron and lipid metabolism is likely to have wide physiological consequences because in all tissues, cellular iron levels interact with lipid metabolism and redox pathways to determine local ferroptotic stress (63).

We discovered that chronic ferroptotic stress in the liver drives senescence in zone 2 hepatocytes — the very subpopulation with the greatest phenotypic plasticity in health. Liver zonation is established by perfusion gradients that shape nutrient, hormone, and metabolite exposure (64). Injury-induced alterations in blood flow can trigger phenotypic shifts in surviving cells by changing their local environment. The mechanisms coordinating these perfusion shifts are not well defined but are critical to understand because they govern changes in liver cell states, function, and regenerative capacity.

Our results indicate that senescent hepatocytes are major regulators of vascular remodeling both within the liver and systemically. As hepatocytes become senescent under ferroptotic stress, their SASPs evolve. We observed that Smo-KO hepatocytes express less VEGF-α, while zone 2 sinusoids lose key LSEC markers (Lyve-1, Emcn2). At the same time, the hepatocyte secretome becomes enriched in Agt, activating the RAS and altering vascular tone and blood flow (65). In vivo imaging of Smo-KO mice confirmed that hepatocyte ferroptotic stress disrupts liver and kidney blood flow. Importantly, Fer-1 treatment rapidly restored hepatic perfusion and normalized sinusoidal markers, while also reducing senescent cell burden and improving liver injury, inflammation, and fibrosis. These observations support the conclusion that hepatocyte ferroptotic stress and the resulting SASPs are major drivers of liver aging.

Although hepatocyte-specific Hedgehog pathway disruption was sufficient to initiate ferroptotic stress and liver aging, hepatocytes likely interact with other organs to determine final outcomes. The increased Agt expression in Smo(–) hepatocytes suggests that the RAS is an important mediator. Hepatocytes are the main source of Agt, and RAS overactivation is a strong risk factor for CKD. RAS inhibitors improve survival in patients with metabolic syndrome-related organ damage (66). Our data in Smo-KO mice provide a plausible mechanistic link between MASLD and renal disease. We showed that selectively deleting Smo in hepatocytes is sufficient to cause kidney injury, fibrosis, and dysfunction, as evidenced by higher Kim-1 expression, increased tubular senescence, macrophage accumulation, interstitial fibrosis, elevated serum creatinine, and decreased renal Epo expression. These findings not only demonstrate that accelerated hepatocyte aging can damage the kidney, they also highlight potential renal signals that may serve as feedback to influence liver regeneration and function.

For example, the kidney is the primary source of circulating Epo. Epo drives erythropoiesis, and RBCs are a major iron reservoir (31). Decreased RBC mass would be expected to perturb systemic iron balance, though we did not directly measure RBC mass in Smo-KO mice and controls — an important goal for future studies. We did, however, observe strongly increased hepcidin immunoreactivity in RTCs of Smo-KO mice, suggesting that disturbed hepatocyte production of iron-regulatory proteins may drive renal hepcidin accumulation and kidney ferroptotic stress. Fer-1 may therefore reverse renal damage by normalizing iron-regulatory protein expression and distribution, reducing ferroptotic stress in both the kidney and liver. Restored Epo production may further benefit hepatocytes. In injured livers, hepatocytes upregulate EpoR, and exogenous Epo protects against MASLD in rodents (40). We observed decreased serum Epo in Smo-KO mice and reduced EpoR mRNA in Smo(–) hepatocytes. This could compromise hepatocyte survival and liver vasculature because, in other contexts, Epo stimulates Smo-dependent signaling and VEGF-α transcription, while VEGF-α supports hepatocyte survival and promotes vasculogenesis (67). Consistent with this finding, we observed decreased VEGF-α and VEGFR2/KDR expression in Smo(–) hepatocytes.

Our work has limitations. We used a model in which aging-related pathology is initiated by hepatocyte-specific Hedgehog pathway disruption. Previously, we also reported that tissue enrichment with the AHGS and SHGS parallels damage progression in other organs that become dysfunctional during systemic metabolic stress, including adipose tissue, heart, skeletal muscle, and kidney (19, 25). Thus, we cannot exclude the possibility that similar mechanisms that accelerate aging might arise in multiple organs. Indeed, emerging evidence suggests that Hedgehog signaling may be suppressed systemically when SMD occurs, given that increased plasma levels of Hedgehog-interacting protein (Hhip), a soluble inhibitor of Hedgehog ligands, have been noted in patients with SMD (68). Importantly, elevated Hhip levels correlate with truncal adiposity, insulin resistance, and other SMD features (69). Evidence that urinary Hhip levels increase before albuminuria in patients with diabetic CKD suggest that reduced Hedgehog signaling may be a trigger for this disease (70). Although the aggregate findings support the premise that progressive deterioration of pathways that maintain cellular plasticity drives biological aging, more work is needed to determine whether SMD and organ dysfunction are consistent consequences of Hedgehog pathway disruption in adults and if so, to delineate the responsible mechanisms.

In summary, our data establish Hedgehog signaling as a key aging-sensitive regulator of ferroptotic stress in hepatocytes and show that hepatocyte ferroptotic stress is a critical determinant of MASLD susceptibility. Because ferroptosis is pharmaceutically tractable (21), these findings have immediate diagnostic and therapeutic implications. They also provide a mechanistic framework to explain why older age increases MASLD incidence, prevalence, and severity (35), and why MASLD is an independent risk factor for other age-related conditions such as type 2 diabetes and CKD (71).

## Methods

### Sex as a biological variable

Our study exclusively examined male mice. It is unknown whether the findings are relevant for female mice.

### Animal studies

#### Phenotypic comparison of control and Smo-KO mice.

Adult male Smo tm2Amc/J (Smo^fl/fl^) mice on a C57BI6/J background (JAX stock 004526; The Jackson Laboratory) were used. At 12 weeks age, mice were injected by tail vein with 5 × 10^11^ genome equivalents of AAV8-TBG-luciferase (control) or AAV8-TBG-Cre recombinase (Smo-KO) to selectively delete Smo in hepatocytes. Vectors were obtained from the University of Pennsylvania Viral Vector Core and Addgene (AAV8-TBG-luciferase [control] #105538-AAV8, AAV8-TBG-Cre #107787-AAV8). Control (*n =* 4) and Smo-KO (*n =* 4) mice were fed a standard chow fed diet and euthanized to harvest liver, kidney, and serum 1 week after vector injection.

#### Hepatocyte isolation and culture studies.

Hepatocytes were isolated from another cohort of Smo^fl/fl^ mice 1 week after injection of either luciferase or Cre vectors (*n* = 4 mice/group) using collagenase perfusion as previously described (72). Some freshly isolated hepatocytes were cultured overnight in standard hepatocyte culture conditions (25) to assess effects of the GPX4 inhibitor, RSL3, on viability. Viability was quantified using CCK8 kit (Dojindo). Results in cultures treated with RSL3 (0.5 μmol/L, MedChemExpress) were compared with vehicle-treated cultures. Other hepatocytes were processed for analysis with either bulk RNA-Seq (*n* = 4 mice) or single-cell RNA-Seq (*n* = 2 mice).

#### Effect of Smo-KO on MASLD and treatment with Fer-1.

To examine the impact of Smo on MASLD susceptibility, additional control (*n =* 9) and Smo-KO (*n =* 17) mice were fed with a CDA-HFD diet (A06071302, choline-deficient, L-amino acid defined diet with 60% kcal fat; Research Diets) for 6 weeks. Vectors were injected by tail vein 1 week before euthanization. For the exploration of ferroptosis’s role in MASLD progression, Smo-KO mice received i.p. injections of 10 mg/kg of Fer-1 (Cayman Chemical) or its vehicle every other day over the last 8 days of the study (*n =* 8) and Fer-1 (*n =* 9). At the end of diet administration of CDA-HFD and treatment with Fer-1, mice were euthanized, blood was obtained, and liver and kidney tissues were fixed in phosphate-buffered formalin for histological analysis, flash-frozen in liquid nitrogen, and stored at –80°C.

### Histological analysis

Liver and kidney samples were fixed in formalin, embedded in paraffin, and sectioned. The sections were subjected to various staining techniques for histopathological evaluation. Sirius red (Sigma-Aldrich, 365548) staining was utilized to evaluate fibrosis, following the manufacturer’s instructions.

For IHC, the slides were dewaxed, hydrated, and treated with 3% hydrogen peroxide for 10 minutes to block endogenous peroxidase activity. Antigen retrieval was performed by heating the slides in 10 mmol/L sodium citrate buffer (pH 6.0) for 10 minutes. Slides were then blocked with Dako protein block solution (Agilent) for 1 hour and incubated overnight at 4°C with specific primary antibodies (Supplemental Table 1) Polymer HRP secondary antibodies were applied for 1 hour at room temperature, followed by detection using the Dako 3,3’-Diaminobenzidine Substrate Chromogen System.

Frozen tissue samples were also used. Sections were cut at a thickness of 20 μm, fixed with 10% formalin, and stained with Oil Red O (Sigma-Aldrich, O0625) for 15 minutes to visualize lipid accumulation. Cellular senescence in the liver was evaluated by senescence-associated β-gal staining using a commercially available kit (Cell Signaling Technology, 9860), following the manufacturer’s instructions. Images were acquired and processed using Leica Microsystems.

### ELISA

The concentration of ferritin and hemopexin in the serum of mice was analyzed by ELISA. Their concentrations were measured using mouse ferritin (Abcam, ab157713) and mouse hemopexin (Abcam, ab157716) ELISA kits in strict accordance with the manufacturer’s instructions and detected using a microplate reader (Tecan) at 450 nm.

### RNA-Seq analyses

#### Bulk RNA-Seq.

Bulk RNA-Seq was done with samples from the Duke MASLD cohort (GSE213623), which includes 368 samples, comprising 69 individuals with obesity but without MASLD, and 299 individuals with obesity and biopsy-confirmed MASLD. Raw FASTQ files were aligned to the hg38 reference genome using STAR (v2.7.4), and gene-level counts were generated with Subread. Low-count genes were filtered, and a normalized count matrix was produced using DESeq2 (v1.38.3). This matrix was then used to generate boxplots in R (v4.4) with ggplot2.

Single-cell RNA-Seq data from our previously published mouse dataset (GSE213183) (11) and human MASLD dataset (GSE330266) (25) were analyzed using Seurat (v5). Raw count matrices from different samples were used to create individual Seurat objects, which were merged, and low-quality cells were filtered out based on mitochondrial gene content (>10%) and feature counts (<200). The data were normalized, and variable features were identified, scaled, and processed through principal component analysis, UMAP, and clustering. To correct for batch effects between samples, Harmony integration was applied. A total of 10,572 cells (mouse dataset) and approximately 68,000 cells (human dataset) were retained for downstream analysis. Gene expression density was visualized using Nebulosa, which performs kernel gene-weighted density estimation to represent gene expression. Cells coexpressing Igfbp2, Cyp8b1, and Hnf4a were classified as zone 2 marker–positive cells; all others were considered zone 2–negative cells. Violin plots were used to compare the expression of genes such as Agt between the experimental groups and zonation categories; statistical significance was assessed using Wilcoxon’s rank-sum test.

### O-link proteomics

Serum protein levels from 80 patients (*n =* 40 with fibrosis stage 0/2 and *n =* 40 with fibrosis stage 3/4) were assessed using Olink Target Panels for cardiometabolic, inflammation, neurology, and oncology. Protein abundance was quantified using normalized protein expression, as previously published (https://www.jci.org/articles/view/180310#sd). Differentially expressed proteins between the 2 groups were identified using the OlinkAnalyze package, applying a Benjamini-Hochberg adjusted *P* value threshold of 0.05. Proteomic data were visualized using ggplot2, and customized functions were from OlinkAnalyze.

### Ultrasound-based 3D hemodynamic imaging

In vivo mouse imaging was performed using a custom ring array–based ULM platform combined with multimodal optical imaging. Gas-filled microbubbles were injected to visualize hepatic and renal flow information and vascular structure, which were quantified from reconstructed ULM maps. For label-free analyses, liver sections were imaged by multimodal optical microscopy to assess metabolic and structural features. Full experimental details are provided in the Supplemental Methods.

### Statistics

Data are expressed as mean ± SEM. Statistical significance between 2 groups was evaluated using an unpaired 2-tailed Student’s *t t*est; comparisons of multiple groups were assessed by 2-way ANOVA. A *P* value of 0.05 or less was considered significant.

### Study approval

We affirm that our research with human samples was conducted in accordance with the Declarations of Helsinki and Istanbul, and approved by Duke University Health System IRB (Pro00005368), with written informed consent received from all participants. Deidentified liver sections from patients with different stages of liver fibrosis were analyzed. All animal studies were approved by the Duke University IACUC (A200-21-09) and fulfilled NIH and Duke University IACUC requirements for humane animal care.

### Data availability

Data are available in the Gene Expression Omnibus (GEO) under accession numbers GSE213623, GSE213183, and GSE330266. Data are available upon reasonable request. All datasets generated and analyzed for this study will be publicly available upon publication. Values for all data points in graphs are reported in the Supporting Data Values file.

## Author contributions

JHJ, RKD, and SWC contributed equally to this study. JHJ, RKD, and AMD conceptualized the project and designed the experiments. JHJ, RKD, SWC, RY, SHO, ZL, KD, DSU, NW, YX, JL, LS, JTC, JY, and AMD carried out the experiments and/or analyzed the data. JHJ, RKD, JY, and AMD wrote the manuscript with input from all authors. All authors reviewed and approved the final version of the manuscript.

## Conflict of interest

The authors have declared that no conflict of interest exists.

## Funding support

This manuscript is subject to the NIH Public Access Policy. Through acceptance of this federal funding, NIH has been given a right to make the Author Accepted Manuscript publicly available in PubMed Central upon the Official Date of Publication, as defined by NIH.

- NIH R01AA10154 and R56DK134334 and the Duke Endowment (to AMD).
- National Institute of Diabetes and Digestive and Kidney Diseases K01DK135793-01A1 (to KD).
- NIH R01 DK139109, National Science Foundation career award 2144788, American Heart Association Collaborative Science award 25CSA1417550, Eli Lilly research award program, Chan Zuckerberg Initiative grant 2024-349531 (to JY).
- NIH R01GM149976, NIH U01AI167892, NIH R21NS125395, NIH U54DK134301, NIH U54 HL165443, Hellman fellow award, and Sloan research fellow award (to LS).

## Supplementary Material

Supplemental data

Unedited blot and gel images

Supporting data values

## Version 1 05/19/2026

In-Press Preview

## Version 2 07/22/2026

Electronic publication
