# Supplementary material for: Hepatocyte hedgehog signaling controls ferroptosis to alleviate aging-related organ dysfunction
Source: JCI Insight. 2026 May 19;11(14):e207066. doi: 10.1172/jci.insight.207066 (PMC13461174; doi:10.1172/jci.insight.207066)
Supplement: Supplementary file 1 [file jciinsight-11-207066-s372.pdf]

Supplemental methods, figures, and tables

Supplemental methods

Ring array-based ultrasound imaging system

The ring array-based system comprises a 256-channel data acquisition device (Vantage 256, Verasonics, Kirkland, WA) and a customized full-ring transducer array (Imasonics, France) combining two half-rings, each with 256 elements (512 in total), 4-cm radius, 5MHz center frequency, and two-way bandwidth (-6dB) of 60%. During imaging, the anesthetized animal was fixed onto a custom-built, acoustically transparent animal holder to minimize motion. The portion of the body below neck was immersed in temperature-regulated warm water bath. The animal holder was then placed at the center of the ring array and raster scanned using a motorized linear actuator.

Imaging sequence

To increase the frame rate and reduce the amount of data acquired, coherent plane wave compounding was utilized as the scanning strategy. The ultrasound B-mode slice at each elevational scanning position was formed by coherently summing the reconstructed results from 20 equally spacing view angles, with the plane wave at each angle produced by 64 elements. A B-mode frame rate of 500 Hz was achieved by setting a 95us time interval between each plane wave transmission-reception cycle. These 20 angles together scan 360 degrees of the animal body and result in an 3cm-diameter effective FOV. A total of 40 positions (step size = 0.4mm) were scanned in Phase 1 and 50 in Phase 2 and 3 to cover most of the liver. To obtain the blood flow information and vascular structure, gas-filled microbubbles (MBs) were injected right before the scanning starts. For later ultrasound localization microscopy (ULM) processing, 2800 frames were acquired per position, leading to a total scanning time of approximately 12 minutes.

Image reconstruction

2D B-mode images of pixel size 70um at each scanning position were reconstructed using the delay-and-sum algorithm. Again, each B-mode image was formed by coherently summing the reconstructions from 20 view angles. Frames containing strong breathing motions were detected and discarded by using a threshold-based cross-correlation method. To extract MB signals, we employed spatial-temporal Singular Value Decomposition (SVD) clutter filtering in which the cutoff for every 250-frame batch was adaptively determined through the correlation matrix of spatial singular vectors (74). The sum of squared of the clutter filtered data forms the Power Doppler (PD) image, and stacking together the PD image from all 40 or 50 scanned positions gives a 3D volumetric view. The filtered MB images were interpolated to achieve a 1/5 resolution of original resolution. Each interpolated frame underwent 2D normalized cross-correlation with the empirically determined point spread function of the imaging system and localized the center positions of MBs. Using uTrack3D algorithm for MB tracking, the linear assignment algorithm was implemented to link localized MB positions across frames (75). A maximum pairing/linking search radius of 5 pixels was assigned, and a linear motion Kalman filter model was applied to restrict the maximum linking angle to 45 degrees, ensuring robust trajectory estimations. Based on the reconstructed MB trajectories, flow direction and velocity maps were computed, providing function imaging information on hemodynamics.

Vessel quantification and ROI selection

ULM rather than PD images were chosen for blood vessel measurements due to its higher SNR, finer spatial resolution, and better vascular contrast. To mitigate the sparsity effect of ULM tracking, a 5x5 averaging filter was applied to refine vascular delineation and reduce stochastic fluctuations. To estimate the blood volume percentage of the liver, defined by the percentage of the volume of blood-perfused vessels within a specific region-of-interest (ROI), three 45-55mm^3

volumes with minimal overlap at the peripheral of the left medial and lateral lobes were manually selected. The mean blood volume ratio is then calculated as the sum of the binarized ROIs over their total voxel number. The mean blood flow speed within those ROIs was also obtained by using the binarized mask to extract the flow speed values from the ULM flow speed map. Similarly, a 120-220mm^3 ROI, depending on the size of the kidney, was chosen to encapsulate most of the right kidney. The blood volume percentage and mean flow speed for the right kidney were then estimated in a similar way.

Animal preparation for 3D-ULM

Adult male Smo tm2Amc /J ($Smo^{flox/flox}$) mice on a C57Bl6/J background (JAX stock# 004526; The Jackson Laboratory, Bar Harbor, ME) were used at 16 weeks old with an average weight of 20g. The imaging region was shaved, and the mouse's body temperature was maintained at 37°C throughout the experiment. Microbubbles were injected during imaging. Gas-filled microbubbles with a diameter of 0.99+/-0.56 μm (1.00+/-0.20×108 bubbles/mL, Revvity, USA) were injected (retroorbital, I.V. injection) right before the imaging. The total volume of microbubbles injected was less than 100 μL.

Sample preparation for label-free multi-modality imaging

Mouse liver tissue samples were fixed using 4% paraformaldehyde (PFA) and stored in 1× PBS at 4°C when not in use. For imaging, mouse liver tissues were sectioned at 80 μm thickness using a PRECISIONARY Compresstome vibratome. Prepared samples were mounted between 1 mm thick glass slides and number 1.5 thickness cover glass for imaging.

Label-free multi-modality optical imaging

For multimodal optical imaging acquisition, we utilized a custom-modified Olympus upright laser-scanning microscope equipped with a high numerical aperture water immersion objective (25×, XLPLN WMP2, NA 1.05). The system incorporated a picoEmerald laser source (Applied Physics & Electronics) that generated two synchronized beams: a tunable pump laser (780-990 nm wavelength range, 5-6 ps pulse duration, 80 MHz repetition frequency) and a fixed-wavelength Stokes beam (1032 nm, 6 ps pulse width, 80 MHz repetition rate). Both beams were directed into the microscope optical path and transmitted light was collected using a high NA (1.4) oil immersion condenser. A high O.D. shortpass filter (950nm, Thorlabs) was used that would completely block the Stokes beam and transmit the pump beam only onto a Si photodiode for detecting the stimulated Raman loss signal. The output current from the photodiode was terminated, filtered, and demodulated in X with a zero phase shift by a lock-in amplifier (HF2LI, Zurich Instruments) at 20MHz. The demodulated signal was fed into the FV3000 software module FV-OSR (Olympus) to form the image during laser scanning. All SRS images were obtained with a pixel dwell time 20 μs and a time constant of 15 μs.Two-photon fluorescence (TPF) imaging employed an 800 nm pump wavelength at 150 mW to excite both NADH and FAD molecules. Signal acquisition used 4 μs pixel dwell times averaged over 6 frames, with emitted fluorescence signals separated using a dual-channel filter configuration (460 nm for NADH and 515 nm for FAD). Second harmonic generation (SHG) was used to capture type 1-3 collagen images. We utilized the 1031 nm Stokes beam at 500 mW power, with signal averaging across 6 frames and a per-pixel dwell time of 4 μs. The resulting SHG signals were filtered using a 465 nm bandpass filter in the backscattered detection path. (Supplemental figure 9)

Data analysis for multi-modality imaging

<u>Ratiometric analysis</u> Radiometric analysis of multimodal images was performed to visualize and quantify the metabolic changes in mouse liver tissues. After acquisition, TPF and SRS images were processed using custom Python scripts. Binary masks were created from NADH and

saturation fatty acids (SFA) images to isolate the tissue without any background. Lipid unsaturation was defined as:

$$Lipid\ Unsaturation = \frac{I_{USFAs}}{I_{USFAs} + I_{SFAs}}$$

where I_{USFAs} and I_{SFAs} correspond to pixel intensities from SRS images at 3012 cm^{-1} (unsaturated fatty acids) and 2880 cm^{-1} (saturated fatty acids), respectively.

For statistical analysis, radiometric values from multiple regions of interest were compiled into pandas DataFrames.

Hyperspectral analysis Hyperspectral SRS (hSRS) imaging was performed by sequentially tuning the pump wavelength from 781.3 nm to 806.5 nm in 60 steps, corresponding to Raman shifts between 2700 cm^{-1} and 3100 cm^{-1} (C-H stretching region). This generated a 3D image stacks (x, y, spectra) with each pixel containing a unique C-H Raman spectrum. Hyperspectral image stacks were reshaped from 3D (x, y, spectra) to 2D (pixels, spectra) arrays using custom Python scripts. Then each pixel's spectra underwent baseline correction to remove water background. Spectra were then normalized using maximum intensity normalization and smoothed with a Whittaker filter (λ=0.2). The scikit-learn implementation of k-means clustering was applied to segment pixels based on spectral similarities. Resulting cluster labels were reshaped back into original image dimensions to create false-color segmentation maps. Mean spectra with standard deviations were plotted for each cluster to identify chemical composition features.

Supplemental Figure 1

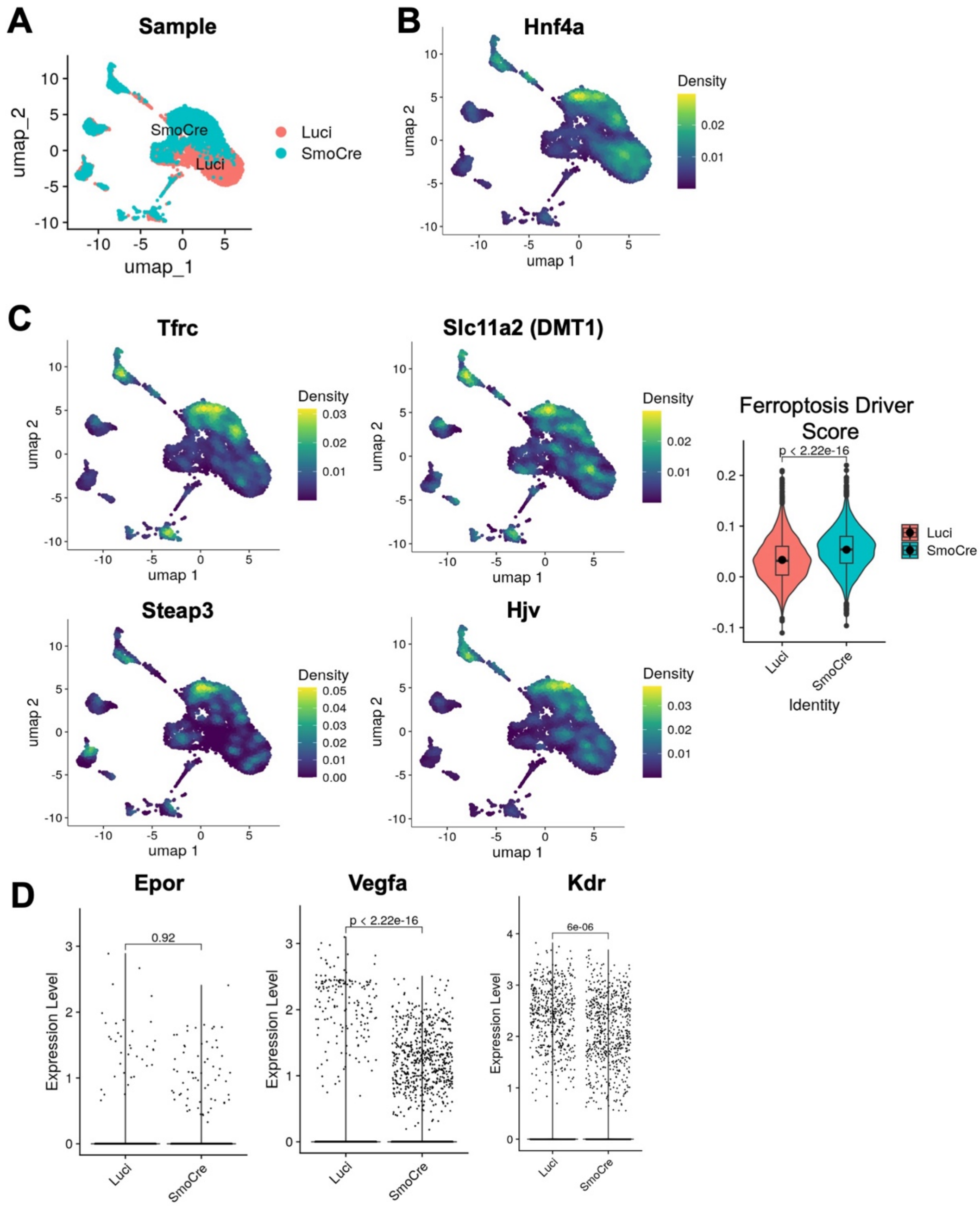

Supplemental Figure 1. Smo depletion in hepatocytes aggravates ferroptotic stress. (A-C) UMAP showing cells from Luci and SmoCre samples (A), hepatocyte marker (B), and ferroptotic stress markers such as tranfrerrin receptor (Tfrc), Slc11a2 (DMT1), Steap3, and Hemojuvelin (Hjv) in control and Smo KO mice hepatocytes and Vln plot showing increased ferroptotic stress score in SmoCre (C). **(D)** VlnPlot showing expression of Epor, Vegfa and Kdr in SmoCre and Luci hepatocytes.

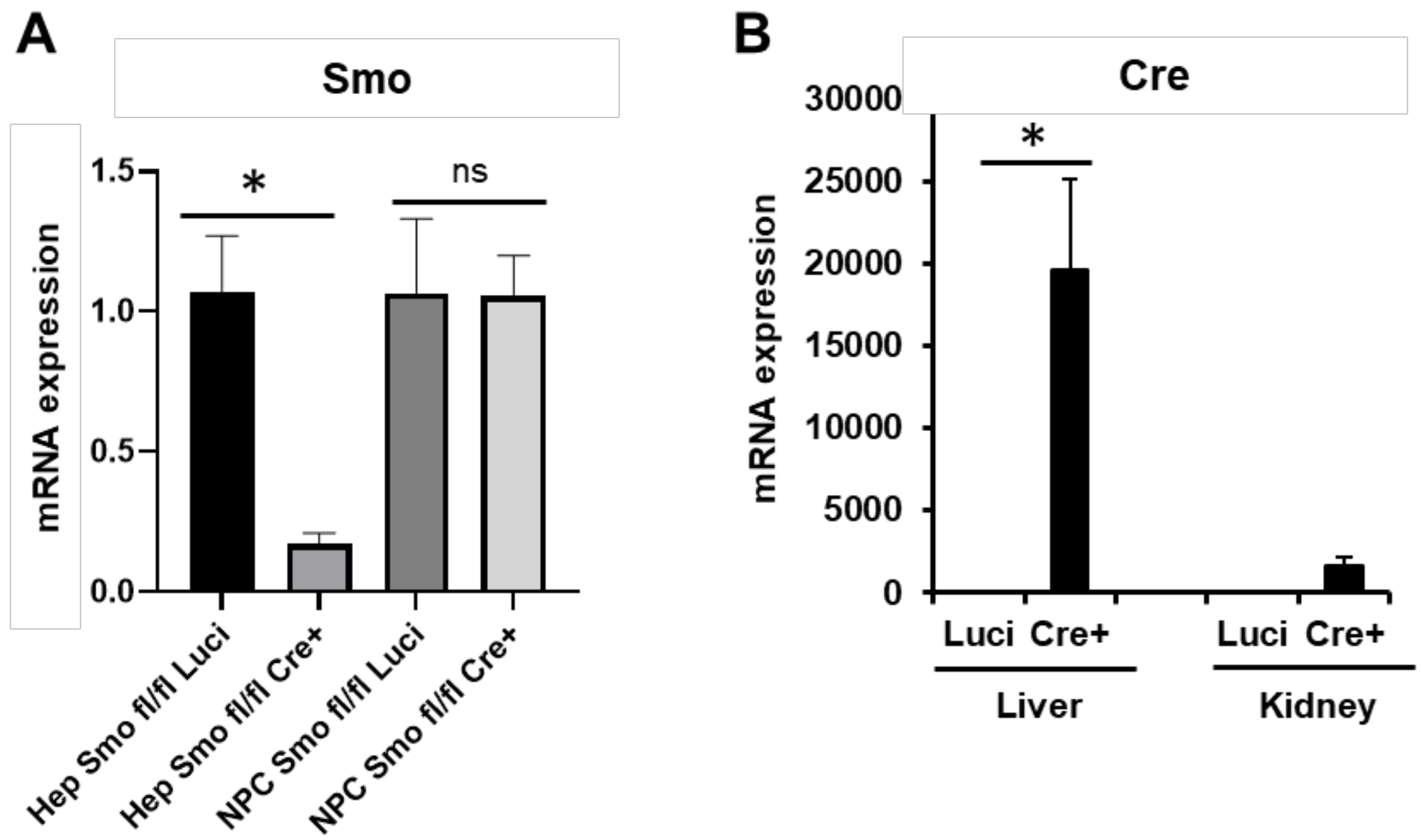

Supplemental Figure 2. AAV8-TBG-Cre selectively depletes Smo expression in hepatocytes without affecting the kidney. Quantitative RT-PCR analysis of Smo and Cre mRNA expression in liver and kidney tissues from AAV8-TBG-Cre–injected Smofl/fl mice and control mice injected with AAV8-TBG-luciferase. **(A)** Smo expression is markedly reduced in liver but not in kidney, confirming hepatocyte-specific deletion. **(B)** Cre expression is detected in liver but not kidney, indicating minimal off-target recombination. Data represent mean ± SEM.

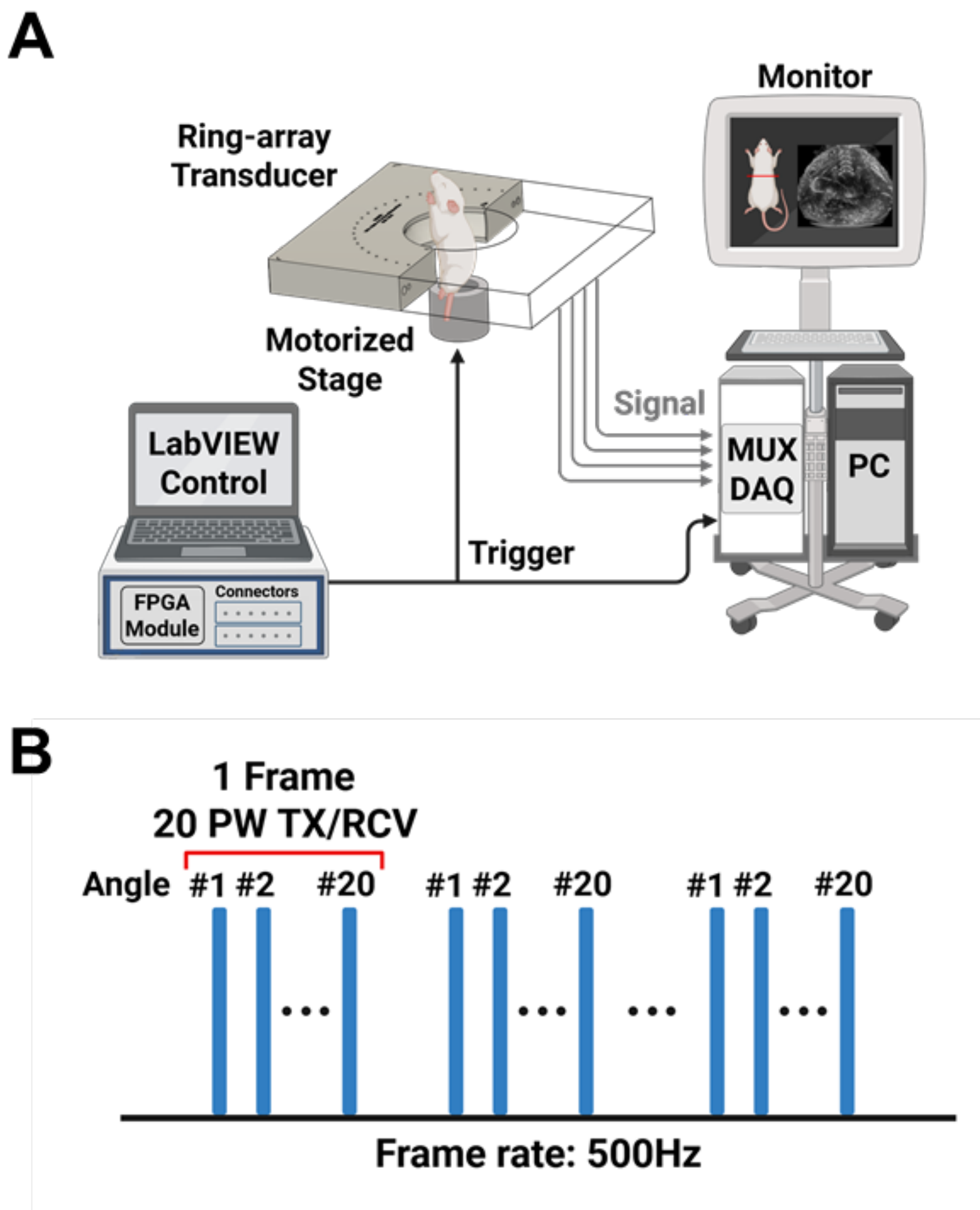

Supplemental Figure 3. Ring-array-based ultrasound localization microscopy (ULM). **(A)** Detailed ring-array ULM system configuration, showing the major system components, including the ring-array ultrasound transduce, the data acquisition system (DAQ), and the FPGA-based system controlling system. **(B)** Transmission/receiving sequence in the ULM system, showing that 20 transmission and receiving events (18° per transmission) are used per image frame, with a frame rate of 500 Hz.

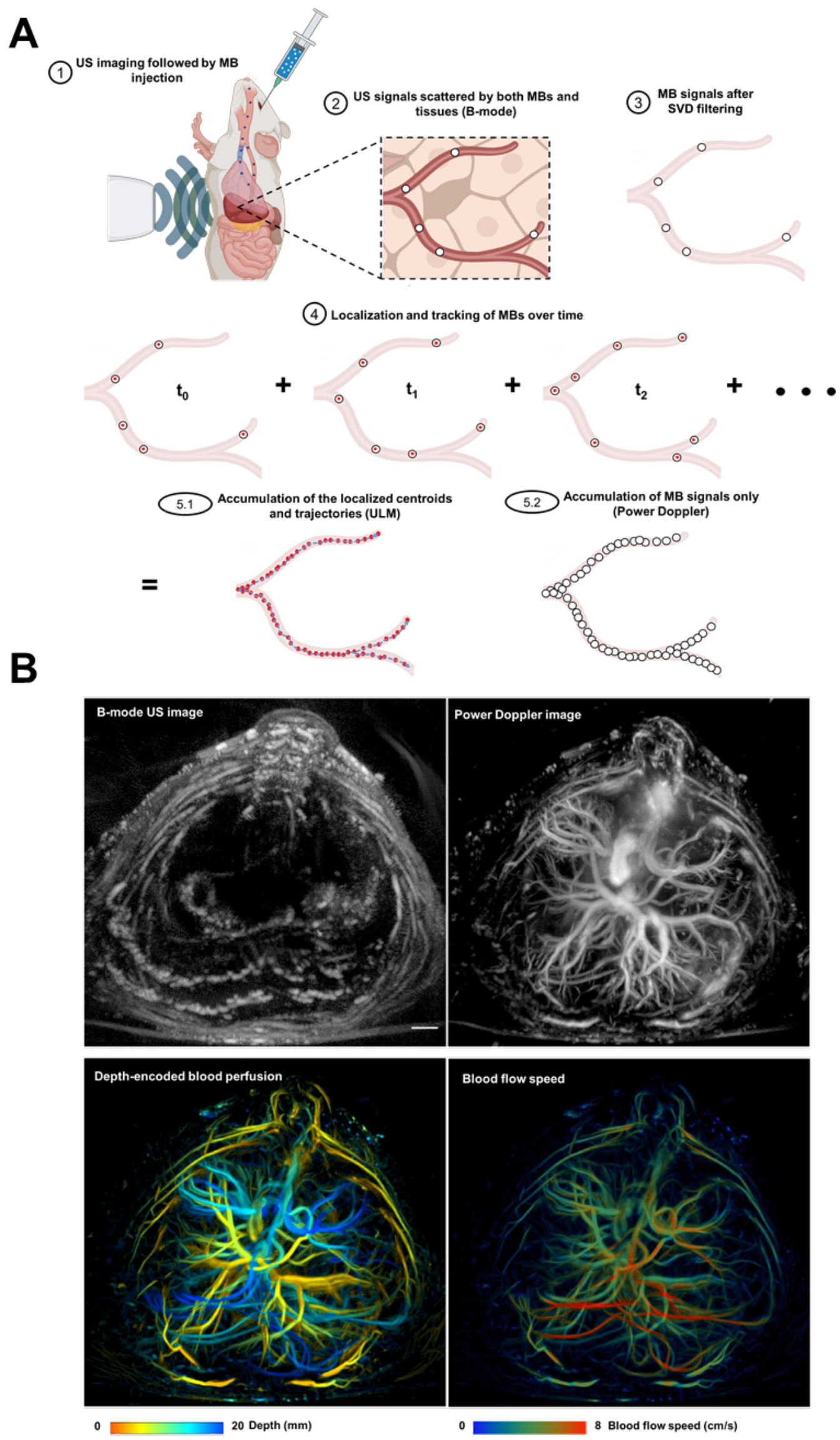

Supplemental Figure 4. The imaging processing in ULM. (A) The key image formation and processing steps in ULM, including the ultrasound B-mode image, the Power Doppler image, and the ULM image. SVD, singular value decomposition. **(B)** Representative images at each key step, showing the liver region blood perfusion and flow speed map.

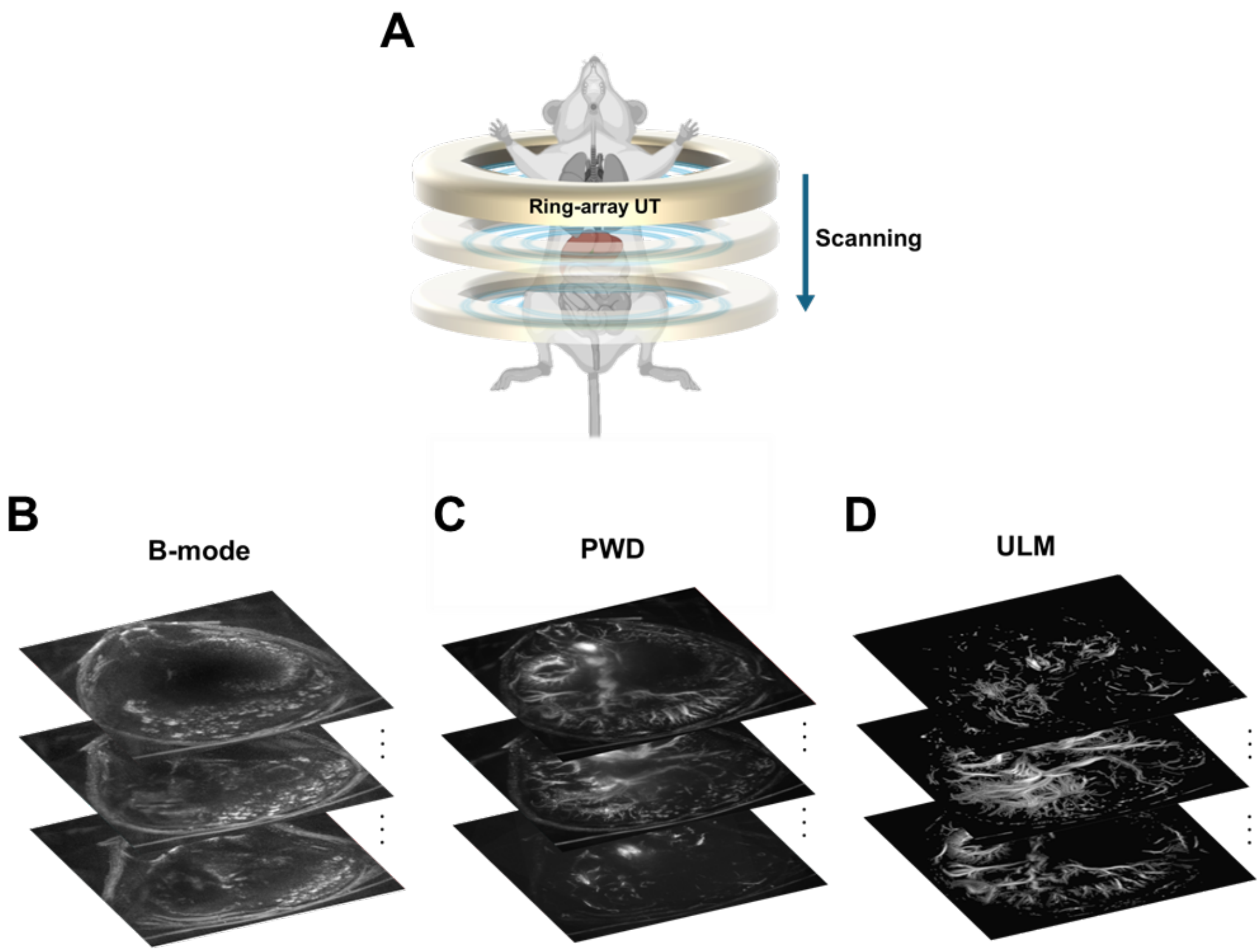

Supplemental Figure 5. 3D ULM image of the mouse at the whole-body level. (A) Elevational scanning scheme of the ring-array based ULM, stepping through each cross-sectional location of the mouse. **(B-D)** Representative stacked cross-sectional images of the B-mode, power Doppler (PWD), and ULM with 2-3 mm section thickness.

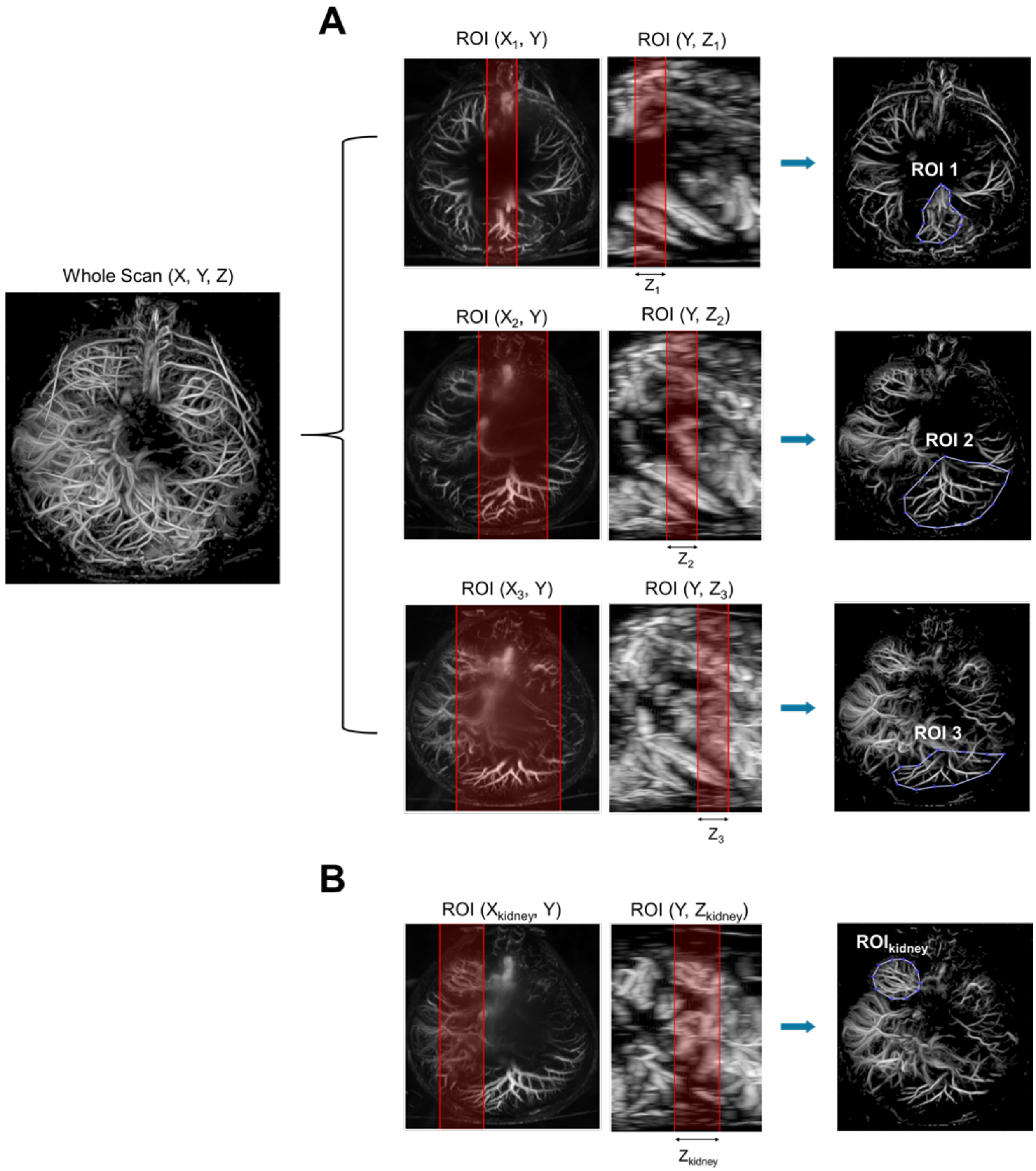

Supplemental Figure 6. Region of interest (ROI) selection for quantitative data analysis in ULM. (A) Representative liver ROI selection. **(B)** Representative kidney ROI selection.

A

B

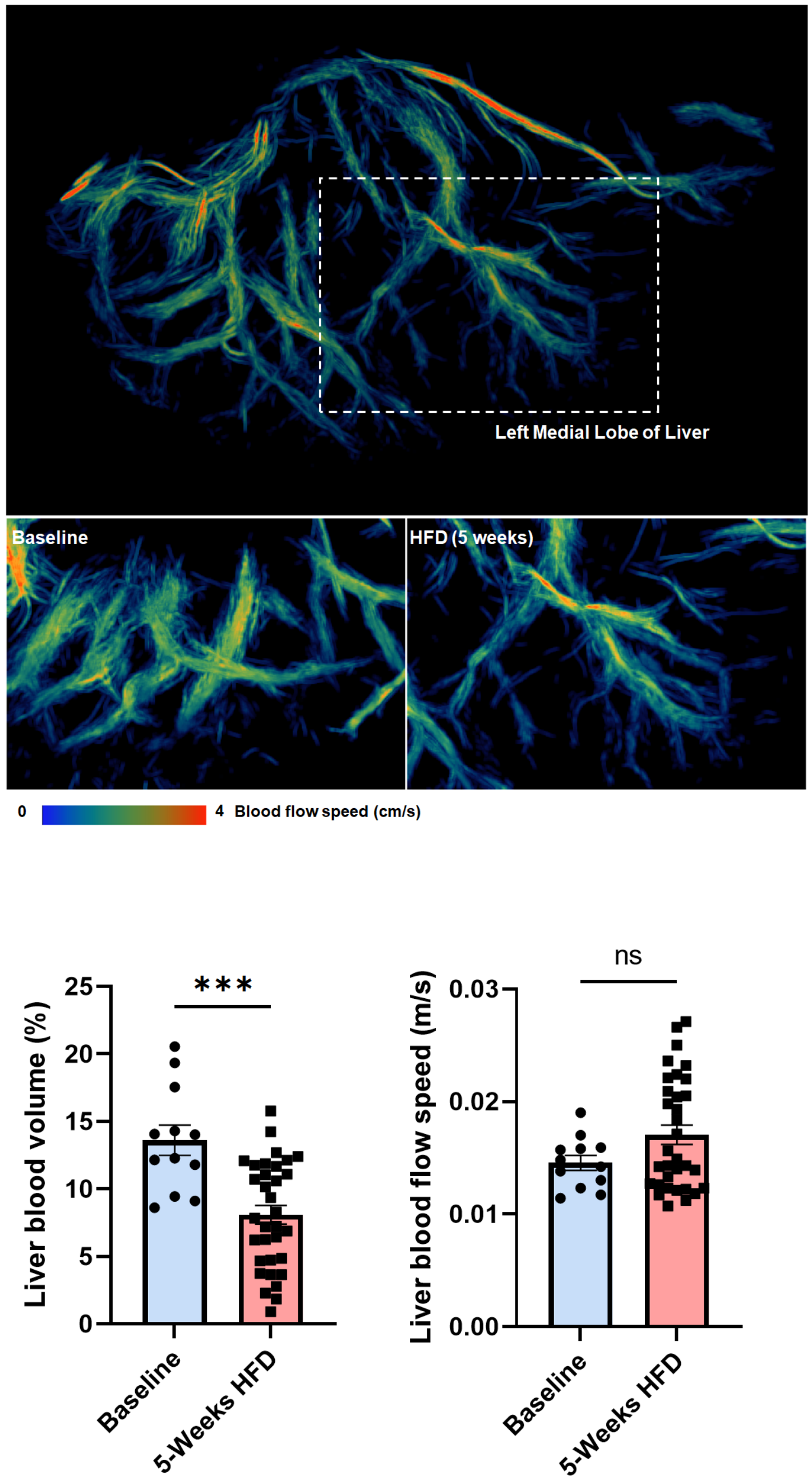

Supplemental Figure 7. Blood perfusion at the liver region before and after the HFD treatment. (A) Representative ULM images of the liver blood flow at Baseline vs. 5-weeks HFD. **(B)** Quantitative analysis of Baseline vs. HFD (blood perfusion, blood flow speed). ***, P<0.001; ns, not significant.

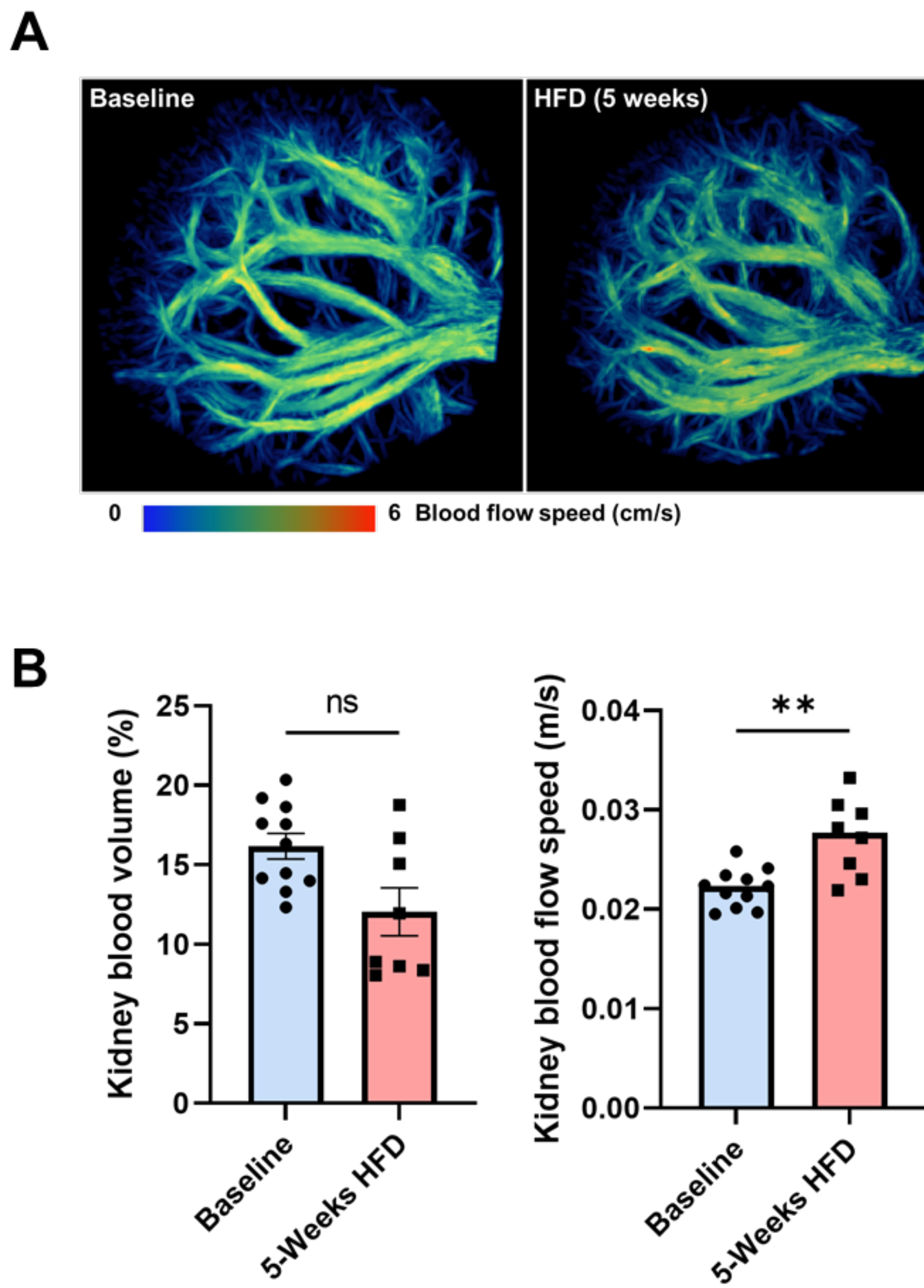

Supplemental Figure 8. Comparing the blood perfusion at the kidney region before and after the HFD treatment. (A) Representative ULM images of the kidney blood flow at Baseline vs. 5-weeks HFD. **(B)** Quantitative analysis of Baseline vs. HFD (blood perfusion, blood flow speed). **, P<0.01; ns, not significant.

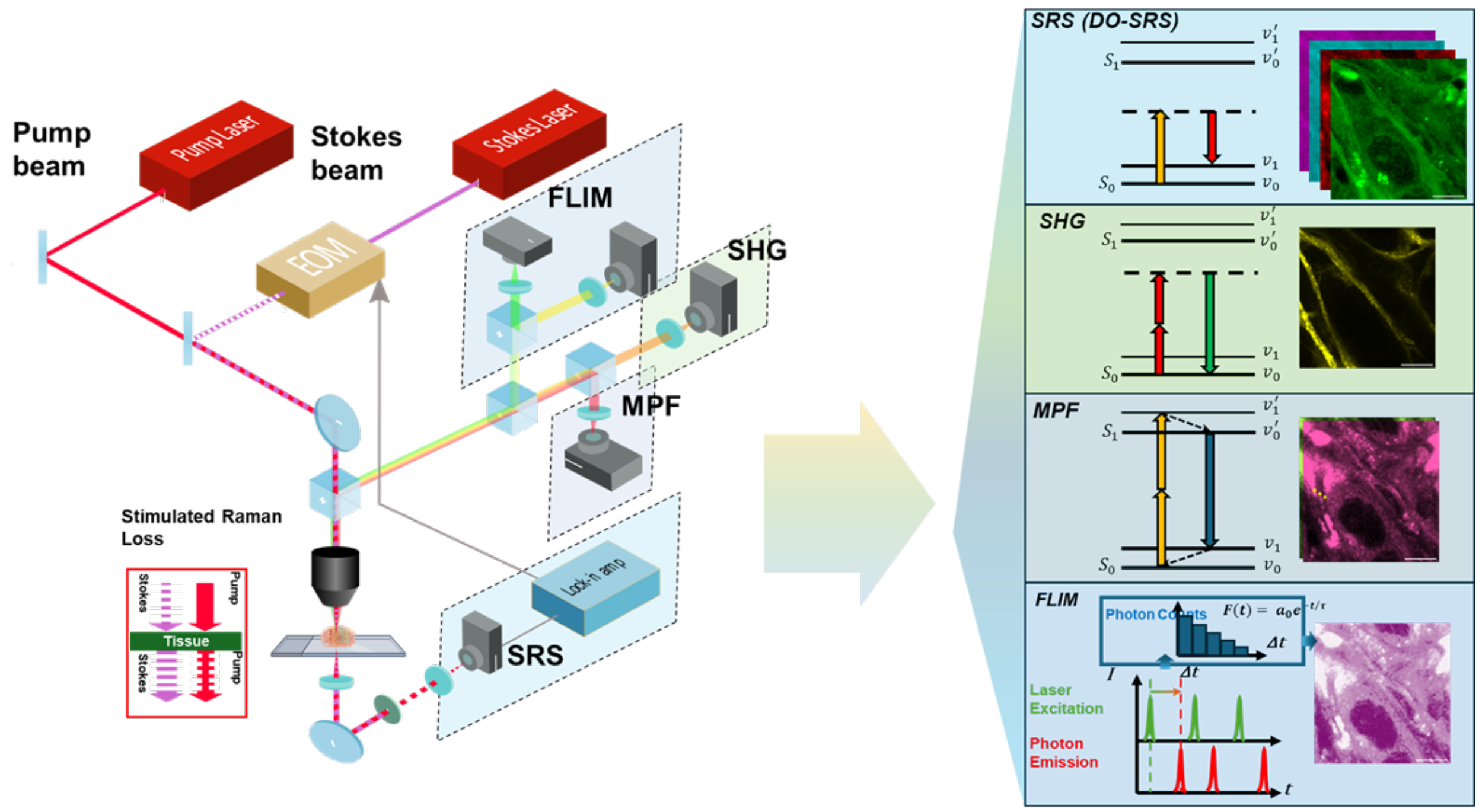

Supplemental Figure 9. Multi-model optical imaging system of the liver tissue samples. A schematic of the home-built multimodal imaging platform in Shi Lab at UCSD: it integrates stimulated Raman scattering (SRS), multiphoton fluorescence (MPF), second harmonic generation (SHG), and Fluorescence lifetime imaging (FLIM) into a single microscopy. All those above-mentioned modalities of signals from various endogenous molecules can be acquired from intact tissues as 2D/3D images in an automatic-co-registered manner.

Supplemental Table 1: Primary antibody used for western blot or immunohistochemistry

Antibody	Host Animal	Dilution	Distributor	Cat. Num.
**ASMA**	Rabbit	1:400	abcam	ab32575
**F4-80**	Rabbit	1:200	Cell Signaling	70076S
**gamma H2A.X**	Rabbit	1:200	abcam	ab81299
**Gli2**	Rabbit	1:400	Aviva systems	OARA02754
**Tfrc**	Rabbit	1:400	abcam	ab84036
**Fth1**	Rabbit	1:1,000	Cell Signaling	3998
**Erythropoietin**	Mouse	1:400	Invitrogen	MA5-43837
**p21**	Rabbit	1:400	abcam	ab188224
**Emcn**	Rat	1:100-	Santa Cruz	sc-65495
**Lyve-1**	RabbitMouse	1:500	Cell Signaling	67538
**Ferroportin (FPN)**	Rabbit	1:200	ThermoFisher Scientific	PA5-22993
**ACSL3**	Rabbit	1:400	Novus Biologicals	NBP2-15252
**ACSL4**	Mouse	1:500	ThermoFisher Scientific	MA5-31548
**Hepcidin (HAMP)**	Rabbit	1:400	Novus Biologicals	NBP1-59337
**Haptoglobin**	Rabbit	1:400	abcam	ab231000
**Glutathione Peroxidase 4 (GPX4)**	Rabbit	1:400	abcam	ab231174
**4-Hydroxynonenal (4-HNE)**	Rabbit	1:500	abcam	ab46545
