# Supplementary material for: Hepatocyte hedgehog signaling controls ferroptosis to alleviate aging-related organ dysfunction
Source: JCI Insight. 2026 May 19;11(14):e207066. doi: 10.1172/jci.insight.207066 (PMC13461174; doi:10.1172/jci.insight.207066)

Original images for Blots

Full western blot gel panels

Figure 2C

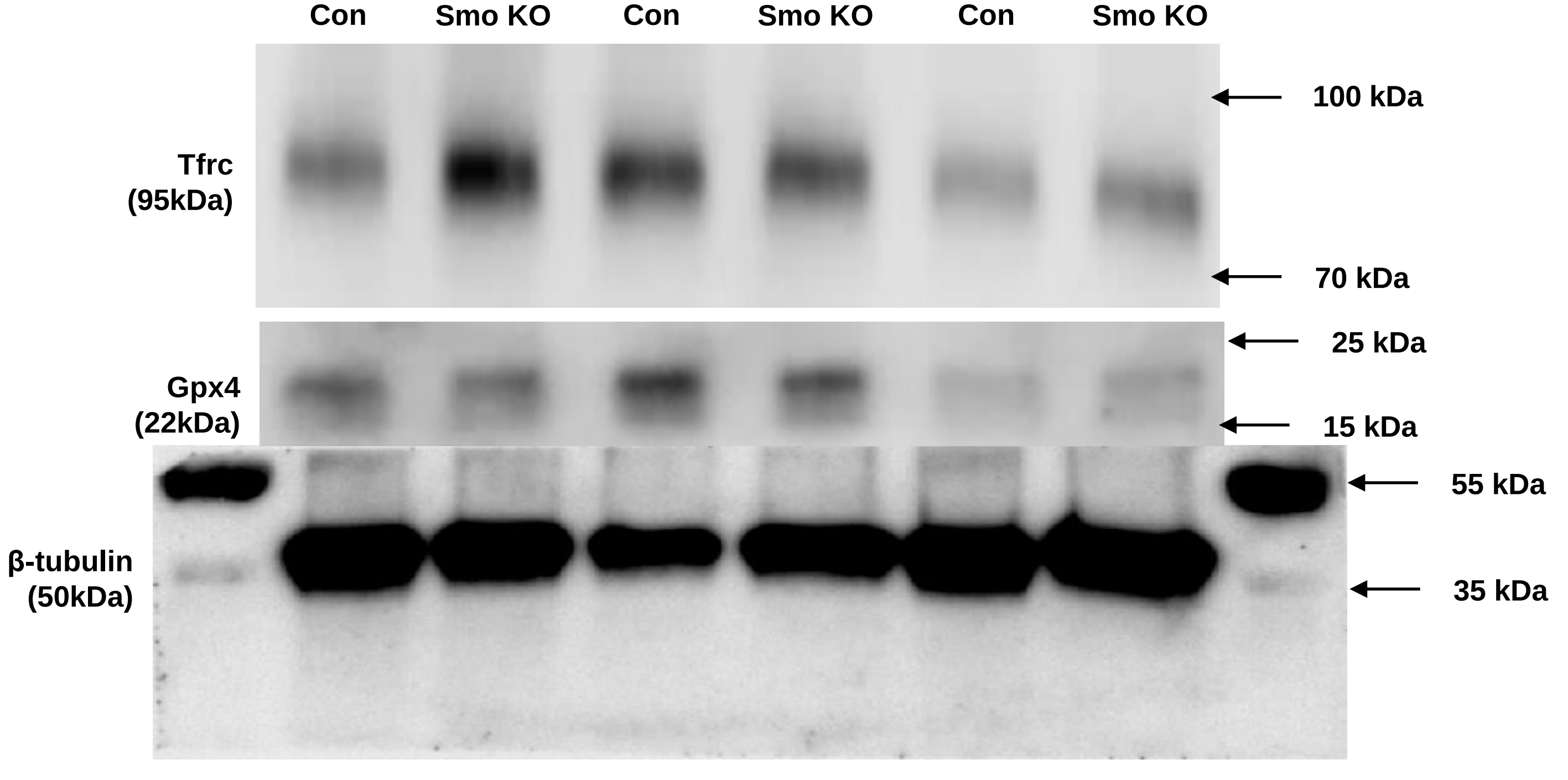

Supplement: Supplementary file 2 [file jciinsight-11-207066-s373.pdf]
